# A conserved juxtacrine signal regulates synaptic partner recognition in *Caenorhabditis elegans*

**DOI:** 10.1186/1749-8104-6-28

**Published:** 2011-06-10

**Authors:** Joori Park, Philip Louis Knezevich, William Wung, Shanté Nicole O'Hanlon, Akshi Goyal, Kelli Leilani Benedetti, Benjamin James Barsi-Rhyne, Mekala Raman, Natalyn Mock, Martina Bremer, Miri Kerensa VanHoven

**Affiliations:** 1 Department of Biological Sciences, San José State University, San José, CA 95192, USA; 2 Department of Mathematics, San José State University, San José, CA 95192, USA

## Abstract

**Background:**

An essential stage of neural development involves the assembly of neural circuits via formation of inter-neuronal connections. Early steps in neural circuit formation, including cell migration, axon guidance, and the localization of synaptic components, are well described. However, upon reaching their target region, most neurites still contact many potential partners. In order to assemble functional circuits, it is critical that within this group of cells, neurons identify and form connections only with their appropriate partners, a process we call synaptic partner recognition (SPR). To understand how SPR is mediated, we previously developed a genetically encoded fluorescent trans-synaptic marker called NLG-1 GRASP, which labels synaptic contacts between individual neurons of interest in dense cellular environments in the genetic model organism *Caenorhabditis elegans*.

**Results:**

Here, we describe the first use of NLG-1 GRASP technology, to identify SPR genes that function in this critical process. The NLG-1 GRASP system allows us to assess synaptogenesis between PHB sensory neurons and AVA interneurons instantly in live animals, making genetic analysis feasible. Additionally, we employ a behavioral assay to specifically test PHB sensory circuit function. Utilizing this approach, we reveal a new role for the secreted UNC-6/Netrin ligand and its transmembrane receptor UNC-40/Deleted in colorectal cancer (DCC) in SPR. Synapses between PHB and AVA are severely reduced in *unc-6 *and *unc-40 *animals despite normal axon guidance and subcellular localization of synaptic components. Additionally, behavioral defects indicate a complete disruption of PHB circuit function in *unc-40 *mutants. Our data indicate that UNC-40 and UNC-6 function in PHB and AVA, respectively, to specify SPR. Strikingly, overexpression of UNC-6 in postsynaptic neurons is sufficient to promote increased PHB-AVA synaptogenesis and to potentiate the behavioral response beyond wild-type levels. Furthermore, an artificially membrane-tethered UNC-6 expressed in the postsynaptic neurons promotes SPR, consistent with a short-range signal between adjacent synaptic partners.

**Conclusions:**

These results indicate that the conserved UNC-6/Netrin-UNC-40/DCC ligand-receptor pair has a previously unknown function, acting in a juxtacrine manner to specify recognition of individual postsynaptic neurons. Furthermore, they illustrate the potential of this new approach, combining NLG-1 GRASP and behavioral analysis, in gene discovery and characterization.

## Background

Neurons are organized into intricate circuits during development by forming synapses with appropriate partners. Electron micrograph reconstruction studies have shown that neurons recognize appropriate synaptic partners despite contacting many other cells (reviewed in [1]). For example, a retinal ganglion axon in the lateral geniculate nucleus forms synapses with only four partners despite contacting 43 cells [2]. In *Caenorhabditis elegans*, the only organism for which there is a complete synaptic map generated through decades of electron micrograph reconstruction studies, on average only one out of six contacting neurons form synapses [3]. However, the molecular mechanisms of synaptic partner recognition (SPR), by which neurons precisely identify only a few 'correct' synaptic partners in the final target region, are poorly understood. The elucidation of these mechanisms is critical to understand the logic of neural connectivity. In this paper we use a new approach, combining the split GFP-based transgenic trans-synaptic marker Neuroligin-1 GFP reconstitution across synaptic partners (NLG-1 GRASP) and a specific behavioral test for circuit function, to study this crucial and final stage in neuronal circuit formation.

The secreted UNC-6/Netrin and its transmembrane receptor UNC-40/Deleted in colorectal cancer (DCC) are a conserved ligand-receptor pair that act in many early steps in neural circuit assembly, including cell migration, axon guidance, dendrite growth, and the localization of presynaptic components. Gradients of UNC-6/Netrin secreted from non-neuronal guidepost cells can regulate cell and axon migration (reviewed in [4-6]). For example, attraction can be mediated by the UNC-40/DCC transmembrane receptor and repulsion by UNC-5 and UNC-40/DCC co-receptors or UNC-5 alone. UNC-6/Netrin can also promote dendrite outgrowth through the UNC-40/DCC receptor [7]. In addition, UNC-6/Netrin can orchestrate the targeting of presynaptic components to subcellular compartments. For example, presynaptic components are localized to the correct neurite domain of AIY interneurons in *C. elegans *via UNC-6/Netrin secreted by sheath cells [8]. Conversely, presynaptic components are excluded from the dendrites of DA9 motorneurons by secreted UNC-6/Netrin [9]. The attractive UNC-6/Netrin signal acts via UNC-40/DCC while the repulsive signal employs UNC-5.

Localizing a neurite to the correct target region and specifying that region of the neurite as synaptogenic may be sufficient to generate correct SPR if the target region is composed solely of correct partners. For instance, in the HSNL motorneuron in *C. elegans*, localization of presynaptic components to the vulval region via immunoglobulin superfamily (IgSF) proteins results in synaptogenesis with vulval muscle cells and the VC neurons likely due to the paucity of other partners in the region [10,11]. However, most neurons contact many different processes within a target region, so that this strategy alone would not be sufficient to identify the correct synaptic partner. Thus, neurites in complex regions must complete neural circuit formation by faithfully recognizing and forming synapses with a small subset of correct partners from the pool of cells within the target region. This final step likely involves a direct interaction between pre- and postsynaptic partners.

Many studies have focused on cell adhesion molecules sufficient to promote synaptogenesis *in vitro *(reviewed in [1]). For example, Netrin-G proteins, vertebrate Netrin-family proteins tethered to the membrane by a carboxy-terminal glycosyl phosphatidylinositol (GPI) anchor, have synaptogenic roles affected by binding transmembrane netrin-G-ligands (NGL-1 to -3) [12-14]. Similar properties have been found for the leucine-rich repeat proteins LRRTM1 and LRRTM2 [15], the IgSF protein SynCAM [16] and others (reviewed in [1]). However, robust *in vivo *phenotypes have not yet been reported for these signals [13,15]. Notably, *in vivo *studies demonstrate that Sidekick-1 and -2 and Dscam and DscamL IgSF transmembrane proteins mediate homophilic interactions that specify lamina-specific targeting of presynaptic interneurons and postsynaptic retinal ganglion cells in the vertebrate retina. However, their ability to trigger synaptogenesis has not yet been explored [17,18].

Electron microscopy is a powerful tool to identify synaptic partners; however, analysis of a single animal requires months to years. Thus, identifying SPR mutants or assaying statistically significant sample sizes for partially penetrant mutants and transgenic animals is impracticable. A fluorescent marker is needed for rapid analysis of large enough samples. However, conventional pre- and postsynaptic markers do not provide the necessary resolution to identify synaptic partners in dense synaptic regions.

To address these limitations, we previously developed a transgenic trans-synaptic fluorescent marker called NLG-1 GRASP that permits instant assessment of correct SPR in living animals [19]. Split-GFP moieties are fused to NLG-1, a protein that localizes to both pre- and postsynaptic sites but does not itself affect synaptogenesis in the neurons tested. By expressing these markers using cell-specific promoters, we can visualize synapses made by defined pre- and postsynaptic partners [19]. Mutations in the few genes known to regulate SPR disrupt NLG-1 GRASP fluorescence even in mutants with normal neurite adhesion, indicating that NLG-1 GRASP labels synaptic connectivity and not neurite adhesion [19].

In this paper, we employ this new technology to study a central question of neural development: how do neurons recognize the appropriate partners? This enables the identification of mutants in which axons correctly migrate to the final target region and recruit synaptic components to the correct compartment, but fail to identify and form synapses with appropriate partners. We employ PHB sensory neurons, which faithfully adhere to and form synapses with AVA interneurons in the *C. elegans *posterior ventral nerve cord despite contacting over 30 other neurites [20], as our model for SPR. Using NLG-1 GRASP and a PHB circuit-specific behavioral assay to probe synaptic function, we provide structural and functional evidence that limiting amounts of UNC-6 act in a juxtacrine signal from postsynaptic AVA neurons to presynaptic PHB neurons to promote PHB-AVA synaptogenesis. Presynaptic PHB neurons receive this signal via the UNC-40 receptor. Unlike earlier steps in circuit formation, such as axon guidance or localization of synaptic components, when UNC-6/Netrin is usually expressed in guidepost cells, we demonstrate a local role for UNC-6 in the postsynaptic neuron itself to specify it as the correct partner. These results demonstrate a novel role for UNC-6/Netrin and UNC-40/DCC in SPR.

## Results

### NLG-1 GRASP allows instant visualization of synapses between PHB and AVA neurons in *C. elegans*

**Figure 1:**
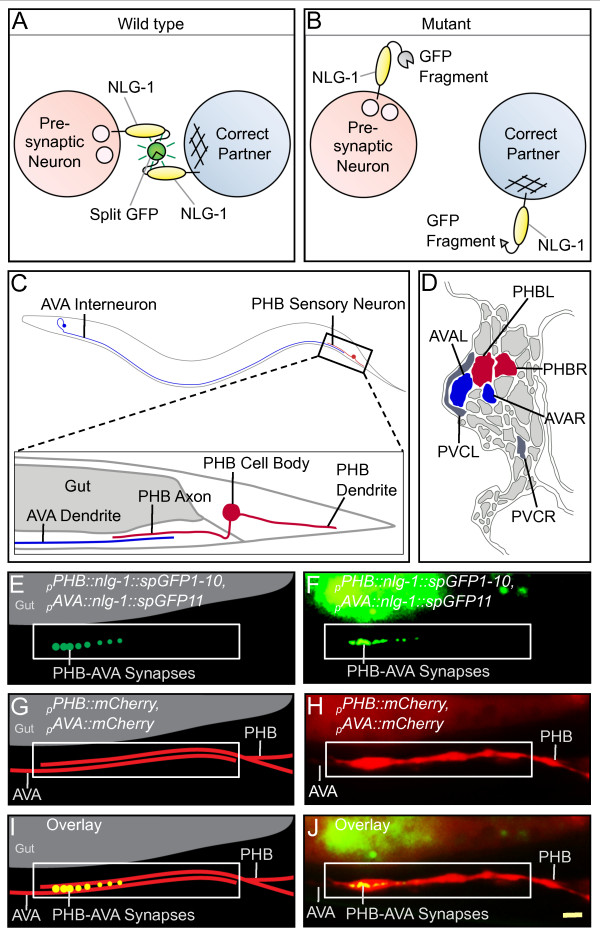
**NLG-1 GRASP labels specific synapses between PHB sensory neurons and AVA interneurons**. **(A) **Schematic diagram of NLG-1 GRASP, which uses split GFP to label synapses formed correctly between pre- and postsynaptic neurons. **(B) **If a neurite fails to identify the correct partner, NLG-1 GRASP will not reconstitute. **(C) **The PHB sensory neurons form synapses with the AVA interneurons in the preanal ganglion (one neuron from each pair is depicted for simplicity). **(D) **Schematic of an electron micrograph representing the preanal ganglion in cross section from a gravid adult, displaying PHB neurons (red) and their primary postsynaptic partners AVA (blue) and PVC (gray) (adapted from [20]). **(E,F) **Schematic (E) and micrograph (F) of the NLG-1 GRASP marker labeling synapses between PHB and AVA neurons. The signal in the dorsal region is gut autofluorescence. **(G,H) **Schematic (G) and micrograph (H) of mCherry-labeled PHB and AVA neurites in contact. **(I,J) **Schematic (I) and micrograph (J) of merged image. White boxes indicate the region of neurite overlap. Yellow scale bar: 2 μm.

We utilized NLG-1 GRASP to visualize synapses between the two PHB sensory neurons and the two AVA interneurons. PHB-AVA synapses are faithfully formed in a nerve bundle extending posteriorly from the ventral nerve cord with approximately 35 parallel neurites in the preanal ganglion. If PHB neurons correctly identify AVA interneurons as postsynaptic partners (Figure 1A), NLG-1 GRASP fluorescence intensity should be high. If SPR is disrupted, the presynaptic neuron should fail to recognize and form synapses with the appropriate postsynaptic target. This may manifest in different ways: after failing to form synapses with the correct partner, the presynaptic neurite might form synapses with a different cell in the target region, or fail to form functional synapses at all. Either possibility indicates a failure of SPR and should result in reduced or absent fluorescence intensity (Figure 1B).

### PHB and AVA neurites form synaptic connections and adhere precisely

PHB neurons are a sensory neuron pair that faithfully forms synapses in the preanal ganglion with two pairs of interneurons controlling backward and forward movement: two AVA and two PVC neurons (Figure 1C) [20-22]. Although PHB neurons extend processes through the center of a bundle containing approximately 35 neurites, they selectively form the majority of their synapses with AVA and PVC (Figure 1D) [3,20]. In this study we have concentrated on PHB synapses made onto AVA interneurons.

If SPR between PHB and AVA were disrupted, two visual phenotypes would be expected: fewer PHB-AVA synapses and defects in neurite adhesion between PHB and AVA neurites. To visualize PHB-AVA synapses and PHB-AVA neurite adhesion in the same animal, we introduced two transgenic markers: NLG-1 GRASP to mark PHB-AVA synapses, and cytosolic mCherry to label both PHB and AVA neurites. Promoters were selected that drive expression specifically either in PHB (*_p_nlp-1 *[23] and *_p_gpa-6 *[24]) or AVA (*_p_flp-18 *[25] and *_p_rig-3 *[26]) within the preanal ganglion. Transgenes carrying the complementary NLG-1 GRASP markers expressed in PHB (*_p_gpa-6::nlg-1::spGFP1-10*) or AVA (*_p_flp-18::nlg-1::spGFP11*) (Figure 1E,F), as well as those driving expression of mCherry in PHB (*_p_nlp-1::mCherry*) or AVA (*_p_flp-18::mCherry*), were generated (Figure 1G,H). All four constructs were co-injected into wild-type animals and stably integrated into the genome to generate the marker strain *wyIs157 *(Figure 1I,J).

**Figure 2:**
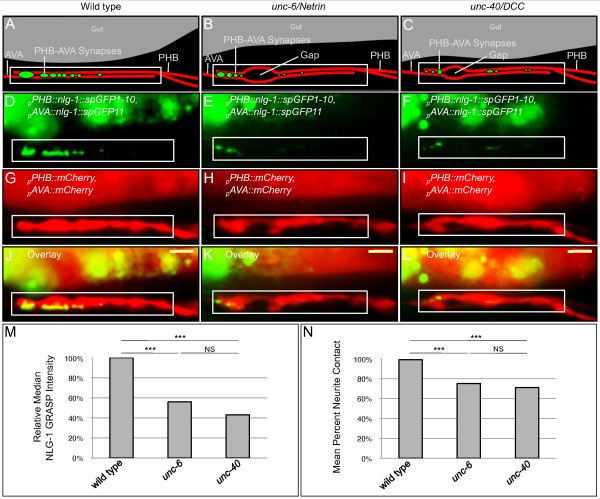
***unc-6 *and *unc-40 *mutants display defective SPR**. **(A) **Schematic and **(D,G,J) **micrographs of a wild-type animal. (D) The NLG-1 GRASP signal indicates synapses between PHB and AVA neurons. (G) mCherry labels PHB and AVA neurites and shows nearly complete contact. (J) Merged image. **(B) **Schematic and **(E,H,K) **micrographs of an *unc-6 *mutant animal. (E) The NLG-1 GRASP signal is severely reduced. (H) Minor defects in neurite contact are visualized as small gaps between mCherry-labeled neurites. (K) Merge. **(C) **Schematic and **(F,I,L) **micrographs of an *unc-40 *mutant. (F) The reduced NLG-1 GRASP signal is similar to that in *unc-6 *animals. (I) Minor defects in PHB and AVA neurite contact are also similar. (L) Merged image. Yellow scale bar: 2 μm. **(M) **Quantification of reduction in NLG-1 GRASP fluorescence in *unc-6 *and *unc-40 *mutants using NIH ImageJ. Wild-type n = 94, *unc-6 *n = 87, and *unc-40 *n = 85 animals. ***P < 0.001, NS, not significant, u-test. *P*-values were adjusted for multiple comparisons using the Hochberg method. **(N) **Quantification of contact defects between PHB and AVA neurites using NIH ImageJ. Wild-type n = 94, *unc-6 *n = 87, and *unc-40 *n = 85 animals. ***P < 0.001, NS, not significant, *t*-test. *P*-values were adjusted for multiple comparisons using the Hochberg method.

In wild-type animals, NLG-1 GRASP puncta are present in the region of neurite overlap, indicating correct synapse formation between PHB and AVA (Figure 2A,D,J). Large bright puncta are frequently present anteriorly, near the end of the PHB axon, while smaller dimmer puncta are frequently found more posteriorly. The degree of PHB-AVA synaptogenesis was quantified using NIH ImageJ (Figure 2M) [27]. Counting puncta is an accurate method for quantifying synapses in sparsely innervated neurons where puncta of approximately the same size and intensity are spread out in a long neurite; however, in short densely innervated neurites such as PHB, large bright accumulations that likely represent several clustered puncta would be counted as a single punctum, resulting in undercounting of synapses. To avoid this, total NLG-1 GRASP intensity was measured using NIH ImageJ. We can visualize SPR early in development (by larval stage L1) and assay animals at early larval stages (L2 to L4).

Labeling both pre- and postsynaptic partners with cytosolic mCherry also permits visualization of extremely subtle contact defects between PHB and AVA neurites that cannot be discerned by labeling only the presynaptic neuron. In wild-type animals, PHB-AVA neurite contact is almost perfect throughout the entire region of neurite overlap in the preanal ganglion, indicating adhesion between neurites (Figure 2A,G,J). The amount of neurite contact was quantified by measuring the lengths of gaps between parallel neurites in the preanal ganglion, or regions where the neurites fail to contact each other, using NIH ImageJ [27]. The percent contact was calculated by subtracting the total length of gaps from the entire length of overlap and dividing by the length of overlap. By this measure, wild-type animals have almost complete neurite contact (Figure 2N).

### *unc-6 *and *unc-40 *mutants have defects in SPR

To identify genes required for correct SPR, the marker *wyIs157 *was crossed into more than 40 candidate mutant strains, including those mutant for cell-adhesion molecules, secreted molecules or transmembrane molecules. The strongest phenotypes were observed for *unc-6 *and *unc-40 *mutants, which lack the secreted UNC-6 protein or its transmembrane IgSF receptor UNC-40, namely the respective orthologs of vertebrate Netrin and DCC.

*unc-6 *and *unc-40 *have well-described defects in axon guidance, although these defects are only partially penetrant. In a minority of mutant animals, at least one PHB axon fails to migrate to the target region, the preanal ganglion, and instead migrates anteriorly along the side of the animal in lateral or subventral positions [28,29]. Animals with axon guidance defects, in which one or both PHB axons fail to reach the preanal ganglion, were not assayed for SPR phenotypes. Only animals without axon guidance defects, in which both PHB neurites reached the preanal ganglion, were assayed for SPR phenotypes.

PHB-AVA NLG-1 GRASP intensity is markedly lower in both *unc-6(ev400) *and *unc-40(e271) *mutants (Figure 2E,F), indicating a defect in synapse formation between PHB and AVA neurons. *unc-6(ev400) *and *unc-40(e271) *likely represent null alleles, as *ev400 *contains a GC-to-AT transition in codon Q78 that introduces a UAA stop codon [30], and phenotypic characterization of *e271 *indicates that it may represent a complete loss of gene function [29]. The fluorescent signal is significantly reduced by approximately 50% in both mutants (Figure 2M), while approximately 50% persists, indicating that other uncharacterized molecules may act in parallel. This indicates that PHB axons are severely impaired in recognizing and forming synapses with their correct partners in *unc-6 *and *unc-40 *mutants.

There are subtle errors in neurite contact within the target region in *unc-6 *and *unc-40 *mutants. Unlike in animals with axon guidance defects, PHB axons reach and enter the target region, the preanal ganglion, and usually contact AVA neurons. However, within the region of PHB-AVA overlap where these neurites normally contact each other almost completely, small gaps are seen between parallel neurites (Figure 2H,I). In *unc-6 *and *unc-40 *mutants, neurite contact is reduced from approximately 97% to approximately 73% (Figure 2N) with no obvious bias for anterior, posterior, or medial gaps (Additional file 1).

To test whether an approximately 25% reduction in PHB-AVA neurite contact is sufficient to result in a loss of synaptogenesis, we examined *unc-7*/*Innexin *mutants [31], which have an approximately 33% reduction in PHB-AVA neurite contact (Figure S2A-B,G-J in Additional file 2). Obviously, a complete disruption in neurite contact would preclude neurons from forming synapses; however, in *unc-7/Innexin *mutants contact is maintained along two-thirds of the region of neurite overlap (Figure S2B in Additional file 2). Strikingly, *unc-7/Innexin *mutants display no defects in NLG-1 GRASP intensity (Figure S2A in Additional file 2), indicating that reducing neurite contact along less than one-third of the overlap region is not sufficient to induce defects in PHB-AVA synaptogenesis. This suggests that the defects in PHB-AVA synaptogenesis and neurite contact observed in *unc-6 *and *unc-40 *mutants are independent consequences of recognition failure between neurites.

Similarly, defects in PHB-AVA synaptogenesis cannot be explained by general defects in nerve bundle fasciculation because mutations in both the *ina-1/α Integrin*, which is required for axon fasciculation in the ventral nerve cord and nerve ring [32], and *sdn-1/Syndecan*, which encodes a transmembrane heparan sulfate proteoglycan required for axon fasciculation in the ventral and dorsal nerve cords [33], do not exhibit defects in PHB-AVA synaptogenesis as measured by NLG-1 GRASP intensity (Figure S2A-B,K-N,O-R in Additional file 2). Thus, the defects in *unc-6 *and *unc-40 *animals are unlikely to result from general nerve bundle defasciculation, but rather reflect a specific failure of SPR between PHB and AVA.

To estimate the proportion of neurite contact required for correct synaptogenesis, the length of each NLG-1 GRASP punctum was measured in wild-type animals and the sum of these lengths was divided by the total PHB-AVA neurite overlap for each animal. Synapses occupy approximately 22% of the total length of neurite contact in wild-type animals (Additional file 3), consistent with the ability of control *unc-7/Innexin *mutants, which maintain contact along approximately 67% of this region, to develop normal PHB-AVA synapses.

To understand if the canonical *unc-6 *and *unc-40 *cell migration or axon guidance pathways also mediate SPR, we examined mutants affecting genes that, in other instances, are either downstream of Netrin signaling (*mig-10/Lamellipodin *[34], *age-1/phosphoinositide 3-kinase (PI3K) *[34], *unc-34/Enabled *[35], and *unc-115/abLIM *[35]) or regulators of Netrin signaling (*unc-5/Unc5 *[29], *unc-129/TGF-beta *[36], and *clec-38*, which encodes a transmembrane protein with C-type lectin-like domains [37]). *unc-34 *animals had highly penetrant defects in PHB and sometimes AVA neurite extension, precluding analysis of SPR phenotype (Figure S4EE-HH in Additional file 4). However, in the remainder of the mutants, no significant SPR defects were observed (Figure S4A-DD in Additional file 4). Interestingly, this suggests that although axon guidance and synaptic partner recognition are mediated by UNC-6/Netrin and UNC-40/DCC, the downstream signaling pathways specifying axon guidance and synaptic partner recognition are likely to be distinct.

### Presynaptic components are found in the correct PHB subcellular compartment in *unc-40 *and *unc-6 *mutants

**Figure 3:**
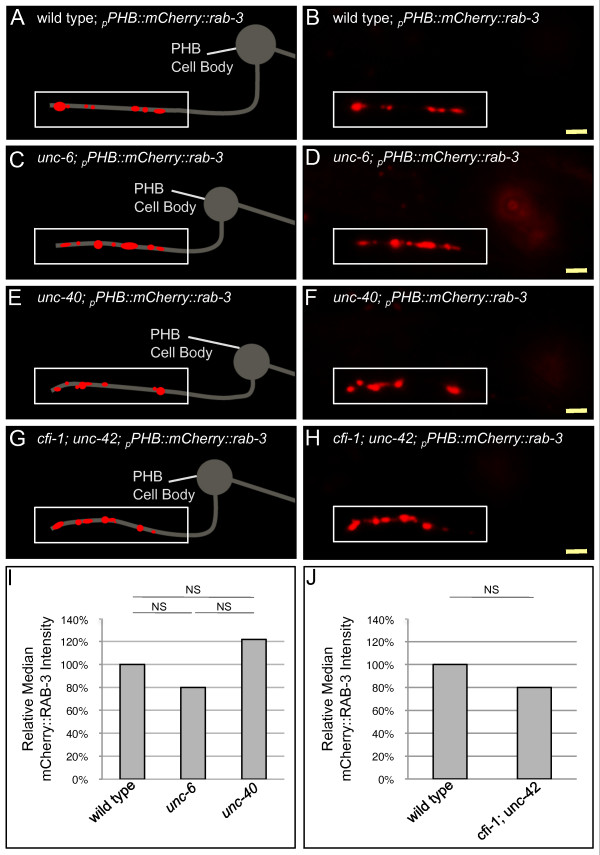
**Presynaptic components localize to the correct subcellular compartment in *unc-6 *and *unc-40 *mutant animals**. **(A,B) **Wild type, **(C,D) ***unc-6*, **(E,F) ***unc-40*, and **(G,H) ***cfi-1; unc-42 *animals are labeled with the presynaptic vesicle marker *mCherry::rab-3 *expressed in PHB neurons. Presynaptic specializations are unaltered in *unc-6*, *unc-40*, and *cfi-1; unc-42 *mutant animals and localize to the distal region of the PHB axon within the preanal ganglion, where PHB synapses normally form (boxed in white). Yellow scale bar: 2 μm. **(I) **Quantification of mCherry::RAB-3 fluorescence intensity using NIH ImageJ indicates no significant difference among wild-type, *unc-6 *and *unc-40 *animals. Wild-type n = 32, *unc-6 *n = 47, and *unc-40 *n = 36 animals. NS, not significant, Kruskal-Wallis test. **(J) **Quantification of mCherry::RAB-3 fluorescence intensity using NIH ImageJ indicates no significant different among wild-type and *cfi-1; unc-42 *animals. Wild-type n = 45, and *cfi-1; unc-42 *n = 45 animals. NS, not significant, u-test.

The failure of PHB and AVA neurons to form synapses correctly may be due to a defect in recognizing the correct synaptic partner or errors in an earlier step of circuit formation, such as the ability to localize presynaptic components to correct neuronal compartments. UNC-6 expression in glia-like sheath cells is required via UNC-40 for correct localization to synaptogenic compartments within neurites of the thermosensation circuit [8]. However, localization of the presynaptic vesicle marker mCherry::RAB-3 to the synaptogenic region of PHB, the distal region of the axon, is normal in *unc-6 *and *unc-40 *mutant animals (Figure 3A-F,3I). Localization of the presynaptic active zone component markers GFP::ELKS-1 and SYD-2::YFP are also normal in *unc-6 *and *unc-40 *mutant animals (Figure S5A-L,S in Additional file 5), suggesting that compartmental localization of presynaptic components is not the primary defect. Similarly, localization of the postsynaptic marker NLG-1::YFP is unaffected in both *unc-6 *and *unc-40 *mutants (Figure S5M-S in Additional file 5), suggesting that trafficking of postsynaptic components is not the primary defect. Since synaptogenesis with the correct partner is disrupted, the synaptic components observed in *unc-6 *and *unc-40 *mutants may represent immature synapses or synapses formed with an incorrect partner, both consistent with a defect in the ability to identify the correct synaptic partner being the primary defect.

To further understand whether normal compartmental localization of presynaptic vesicles is consistent with a failure to recognize the correct partner, we examined the intensity of mCherry::RAB-3 in double mutants affecting the differentiation and/or axon guidance of both primary synaptic partners: AVA and PVC interneurons (Figure 3G,H,J). If postsynaptic partners are not present or differentiated, PHB neurons should be unable to form synapses with the correct partners; we can observe the placement of presynaptic vesicles in these animals. The homeodomain gene *unc-42 *is required for correct differentiation of AVA and axon guidance of AVA neurites into the ventral nerve cord [38,39], and the ARID domain DNA-binding gene *cfi-1 *is required for appropriate differentiation of PVC and AVD interneurons [40]. Strikingly, in *cfi-1; unc-42 *double mutants, mCherry::RAB-3 intensity in the distal region of the PHB axon is normal (Figure 3G,H,J). This indicates that localizing presynaptic components to the preanal ganglion and forming synapses with the correct partner are separable steps in PHB neurons. Identification of the correct synaptic partners is not required for trafficking of presynaptic vesicles from the cell body to the distal PHB axons. In addition, mCherry::RAB-3 intensity in the synaptic region should be normal in SPR mutants, consistent with our findings for *unc-6 *and *unc-40 *animals.

### Cell-specific rescue of UNC-6 in AVA and UNC-40 in PHB neurons

If UNC-6 and UNC-40 mediate a direct interaction between pre- and postsynaptic neurons, these molecules should be expressed in AVA and PHB neurons. Previous studies have reported that UNC-6 is expressed in AVA but not PHB neurons [30] and that UNC-40 is expressed in PHB but not AVA neurons [41]. This differential expression in pre- and postsynaptic neurons suggested to us that UNC-6 and UNC-40 might mediate a direct interaction between pre- and postsynaptic partners. However, both molecules are also expressed in other neurites in the region; UNC-6 is expressed in PVQ, PVT, AVG, VB11, and VA12 while UNC-40 is expressed in PHA, DA8, DA9, VA12, DD6, and VB11 [26].

**Figure 4:**
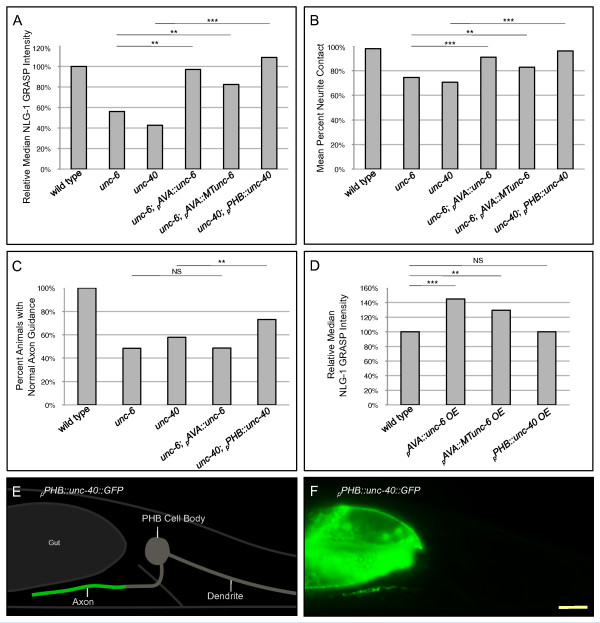
**Expression of UNC-6 in AVA or UNC-40 in PHB is sufficient for SPR**. **(A) **Expression of *_p_AVA::unc-6 *in *unc-6 *mutants and *_p_PHB::unc-40 *in *unc-40 *mutants restores NLG-1 GRASP fluorescence, labeling PHB-AVA synapses. NLG-1 GRASP fluorescence is also significantly rescued in *unc-6 *mutants by expressing a membrane-tethered *unc-6 *in AVA (*_p_AVA::MTunc-6*). ***P < 0.001, **P < 0.01, u-test. **(B) **Neurite contact between PHB and AVA is significantly rescued by expression of *_p_AVA::unc-6 *or *_p_AVA::MTunc-6 *in *unc-6 *mutants and *_p_PHB::unc-40 *in *unc-40 *mutants. ***P < 0.001, **P < 0.01, *t*-test. (A,B) Two lines were examined for each transgene; in all cases, both lines gave similar results. Wild-type n = 254, *unc-6 *n = 87, *unc-6; _p_AVA::unc-6 *n = 70, *unc-6; _p_AVA::MTunc-6 *n = 84, *unc-40 *n = 85, and *unc-40; _p_PHB::unc-40 *n = 79 animals. **(C) **PHB axon guidance to the preanal ganglion is restored by expression of *_p_PHB::unc-40 *in *unc-40 *mutants but not by expression of *_p_AVA::unc-6 *in *unc-6 *mutants. Wild-type n = 94, *unc-6 *n = 180, *unc-40 *n = 147, *unc-6; _p_AVA::unc-6 *n = 144, and *unc-40; _p_PHB::unc-40 *n = 108 animals. **P < 0.01, NS, not significant, χ^2 ^goodness-of-fit test. **(D) **Overexpression of *_p_AVA::unc-6 *and *_p_AVA::MTunc-6 *in wild-type animals increases NLG-1 GRASP fluorescence intensity, indicating an increase in PHB-AVA synapses, while overexpressing *_p_PHB::unc-40 *does not, indicating that the UNC-6 signal is limiting. Two lines were examined for each transgene with the exception of *_p_PHB::unc-40 *overexpression, where three lines were examined; in all cases, all lines gave similar results. Wild-type n = 165, *_p_AVA::unc-6OE *n = 82, *_p_AVA::MTunc-6OE *n = 79, and *_p_PHB::unc-40OE *n = 119 animals. ***P < 0.001, **P < 0.01, NS, not significant, u-test. (A-D) *P*-values were adjusted for multiple comparisons using the Hochberg method. **(E) **Schematic and **(F) **micrograph of UNC-40 subcellular localization. UNC-40::GFP is localized to the region of contact with AVA within the PHB axon, but is excluded from the commissure and dendrite. Yellow scale bar: 5 μm.

If UNC-6 and UNC-40 orchestrate a direct interaction between pre- and postsynaptic neurons, expression of UNC-6 in AVA neurons should rescue the *unc-6 *mutant phenotype and expression of UNC-40 in PHB neurons should rescue the *unc-40 *mutant phenotype. Consistent with these predictions, expression of the *unc-6 *coding sequence under the control of the AVA-selective promoter *rig-3 *rescues NLG-1 GRASP intensity and neurite contact defects in *unc-6 *mutants (Figure 4A,B; Figure S6E-H in Additional file 6). Similarly, the *unc-40 *coding sequence under the control of the PHB-selective promoter *nlp-1 *rescues both SPR phenotypes (Figure 4A,B; Figure S6M-P in Additional file 6).

This model predicts a novel juxtacrine role for UNC-6/Netrin in SPR. If UNC-6 has a local role, a membrane-tethered UNC-6 protein expressed in AVA neurons should rescue the *unc-6 *mutant phenotype. To test this prediction, transgenic animals were generated in which *unc-6 *is fused to the *nlg-1 *transmembrane domain to generate a membrane-tethered *unc-6 *(*MTunc-6*) (D Colón-Ramos, personal communication) and expressed in AVA neurons (*_p_rig-3::MTunc-6*). In fact, PHB-AVA synaptogenesis and neurite contact are significantly rescued by AVA-specific expression of membrane-tethered UNC-6 (Figure 4A,B; Figure S6I-L in Additional file 6), suggesting that UNC-6/Netrin functions at short range to mediate SPR.

Expression of UNC-40 in PHB also significantly reduces the proportion of animals with defects in axon guidance to the preanal ganglion; however, expression of UNC-6 in AVA does not rescue axon guidance (Figure 4C). This suggests that while expression of UNC-6 in AVA is sufficient to rescue SPR, expression elsewhere is required for correct axon guidance to the preanal ganglion. This is consistent with previous work indicating that PHB axon guidance is directed by UNC-6 expression in ventral epidermoblasts, PVT, and PVQ cells [30]. Thus, expression of UNC-6 in AVA regulates SPR while expression in other cells mediates axon guidance.

### Overexpression of UNC-6 in AVA neurons increases the number of PHB-AVA synapses

If UNC-6 functions as a signal from AVA to PHB to promote correct SPR, increasing the expression of *unc-6 *might increase the number of synapses between these neurons. In fact, overexpression of *unc-6 *or *MTunc-6 *in AVA neurons in wild-type animals increases the NLG-1 GRASP intensity significantly above wild-type levels (Figure 4D; Figure S6Q-X in Additional file 6). Thus, UNC-6/Netrin is sufficient to promote PHB-AVA synaptogenesis. However, overexpression of UNC-40 does not have this effect (Figure 4D; Figure S6Y-BB in Additional file 6). Thus, adding UNC-40 receptor without additional ligand does not promote increased synaptogenesis, indicating that *unc-6 *is a limiting factor in SPR between PHB and AVA.

### UNC-40 is localized to the region of the PHB axon that contacts AVA

Localization of UNC-40 along the entire region of contact between PHB and AVA in the preanal ganglion would be consistent with its proposed adhesive role in SPR. To visualize localization of UNC-40 in PHB, UNC-40 was tagged with GFP and expressed specifically in PHB using the PHB promoter *gpa-6 *(*_p_gpa-6::unc-40::GFP*). UNC-40 localizes along the PHB axon in the preanal ganglion where PHB contacts and forms synapses with AVA, and is excluded from other regions of the neuron, including the commissure and dendrite. This is consistent with an adhesive role in SPR (Figure 4E,F). This localization is not changed in *unc-6 *mutants (Figure S6CC-FF in Additional file 6), suggesting that UNC-6 binding to UNC-40 receptors does not specify trafficking or localization of the receptor to the distal axon, but likely acts through a more conventional mechanism, such as activating the receptor and triggering a downstream signaling cascade.

UNC-6 was recently reported to have a punctate pattern in ventral nerve cord neurites, including AVA, a distribution which requires the *unc-104/KIF1A *kinesin motor protein [42]. Consistent with this observation, we found that *unc-104 *mutants had defects in NLG-1 GRASP intensity and neurite contact similar to those displayed in *unc-6 *mutants (Figure S7A-B,H-K in Additional file 7), suggesting a requirement for neurite localization of UNC-6 in PHB-AVA SPR. In addition to the defects in UNC-6 localization, *unc-104 *mutants have well-characterized deficiencies in transporting presynaptic components [43] that likely contribute to the decrease in NLG-1 GRASP intensity. However, the observed decrease in neurite contact is consistent with a requirement for UNC-6 localization. This limited amount of UNC-6 in dispersed puncta in comparison with UNC-40, which is present along the entire region of neurite contact, is consistent with UNC-6 being a limiting factor.

### A behavioral assay for synaptic function between PHB and AVA neurons

**Figure 5:**
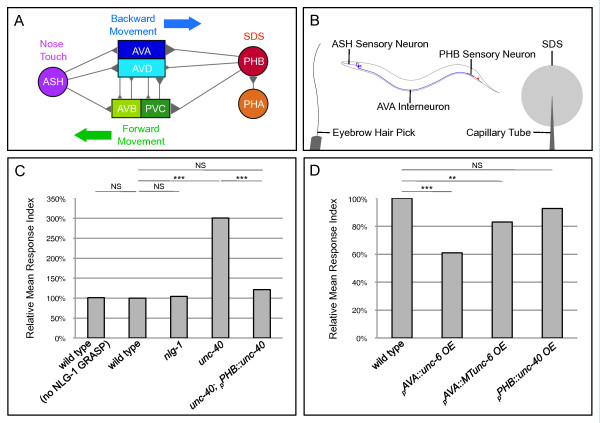
**A PHB circuit-specific behavioral assay indicates that UNC-40 and UNC-6 mediate formation of functional synapses**. **(A) **Neural circuit diagram summarizing synaptic contacts between neurons in PHB and ASH sensory circuits. **(B) **Behavioral assay outline. Function of the PHB circuit is tested by stimulating ASH neurons. ASH-AVA synapses control backward movement induced by a nose touch with an eyebrow hair pick, and PHB-AVA synapses control termination of backwards movement in response to the noxious chemical SDS. **(C) **Neither the NLG-1 GRASP marker nor mutations in *nlg-1 *affect SDS sensitivity. However, *unc-40 *mutants fail to respond to SDS, indicating complete loss of PHB-circuit function. Cell-autonomous expression of *unc-40 *in PHB neurons rescues the behavioral defect. *unc-6 *animals are too uncoordinated to be examined with this assay. Two lines were examined for each transgene; in all cases, both lines gave similar results. Wild-type n = 200, wild-type (no NLG-1 GRASP) n = 40, *nlg-1 *n = 40, *unc-40 *n = 40, *unc-40; _p_PHB::unc-40 *n = 80 animals. ***P < 0.001, NS, not significant, *t*-test. *P*-values were adjusted for multiple comparisons using the Hochberg method. **(D) **Overexpression of *unc-6 *and *MTunc-6 *in AVA neurons potentiates the behavioral response while overexpression of *unc-40 *in PHB does not, indicating that UNC-6 is limiting. Two lines were examined for each transgene with the exception of *_p_PHB::unc-40 *overexpression, where three lines were examined; in all cases, all lines gave similar results. Wild-type n = 200, *_p_AVA::unc-6 *overexpression n = 80, *_p_AVA::MTunc-6 *overexpression n = 80, and *_p_PHB::unc-40 *overexpression n = 80 animals. ***P < 0.001, **P < 0.01, NS, not significant, *t*-test. *P*-values were adjusted for multiple comparisons using the Hochberg method.

If SPR between PHB and AVA is disrupted, function of the PHB sensory circuit should be compromised. Synapses between PHB and its postsynaptic partners AVA and PVC interneurons mediate a worm's ability to stop backing into 0.1% sodium dodecyl sulfate (SDS) detergent (Figure 5A) [44]. We developed a behavioral assay to test the function of this circuit based on an assay initially used by Hilliard and colleagues; animals are touched on the nose using a hair pick to elicit backward movement and a dry drop of SDS is placed behind the animal (Figure 5B; Additional files 8 and 9). Wild-type animals without the NLG-1 GRASP marker back into control buffer for approximately 2.2 seconds but stop backing into SDS after only approximately 0.7 seconds. The average amount of time that an animal spends backing into SDS before stopping is compared to the average amount of time it backs into control buffer, using a response index where the average backing time in SDS is divided by the average backing time in buffer and wild-type is normalized to 100% (Figure 5C).

To determine if the NLG-1 GRASP marker affects synaptogenesis between PHB and AVA, the response of transgene-carrying animals was assayed. If synaptogenesis between PHB and AVA is increased by the NLG-1 GRASP marker, then SDS avoidance should be enhanced in animals with the NLG-1 GRASP transgene compared with non-marker carrying animals. However, wild-type animals expressing NLG-1 GRASP are indistinguishable from wild-type animals without the marker in their response to SDS (*P *= 0.9; Additional file 10), indicating that the marker does not affect PHB-AVA synaptogenesis (Figure 5C). This is consistent with previous results. If NLG-1 GRASP labeling induced additional synapses, it would likely preclude loss of NLG-1 GRASP signal in previously characterized synaptic specificity mutants. However, NLG-1 GRASP labeling of synapses between AVA and VA and DA motorneurons is successfully disrupted in *unc-4 *and *unc-37 *mutants (as previously observed with electron microscopy) [19]. Similarly, if NLG-1 GRASP labeling induced additional synaptogenesis, synapses would likely be induced throughout a neurite's trajectory wherever the postsynaptic partner was present even if electron microscopy studies indicate a smaller discrete region of synaptogenesis. However, the neurites of synaptic partners AFD and AIY traverse the entire nerve ring, but NLG-1 GRASP only labels a discrete dorsal region in which synapses are detected by electron microscopy. Similarly, AIY and RIA traverse the entire nerve ring, but synapses are only labeled in a discrete ventral region, consistent with electron microscopy studies [19]. If NLG-1 GRASP did induce synaptogenesis between PHB and AVA that was undetectable in our assay, it would indicate that the SPR defects in *unc-40 *and *unc-6 *were in fact more severe.

A disruption in SPR between PHB and AVA should result in a failure to sense SDS, namely the backing time in SDS should increase to the same duration as the backing time in buffer and thereby the response index should increase to approximately three times that of the wild-type level. *nlg-1 *mutant animals respond similarly to wild-type animals, indicating that *nlg-1 *is not required for PHB-AVA SPR (Figure 5C). However, the *unc-40 *response index increased to approximately 300%, indicating that *unc-40 *animals do not sense SDS and the PHB circuit is defective (Figure 5C). This is consistent with a decrease in NLG-1 GRASP in *unc-40 *mutants (Figure 2M) and indicates that synaptic function is compromised. *unc-6 *mutants cannot be tested in this assay due to their severely uncoordinated phenotype. In addition, SDS sensitivity was restored by cell-specific expression of *unc-40 *in PHB neurons (Figure 5C). This is consistent with rescue of NLG-1 GRASP intensity by the same transgene (Figure 4A) and indicates that UNC-40 functions in PHB to mediate SPR with AVA neurons.

To test if overexpression of UNC-6 increases synaptic function, SDS sensitivity was tested in wild-type animals expressing the *_p_rig-3::unc-6 *and *_p_rig-3::MTunc-6 *transgenes. Strikingly, the response index decreased in wild-type animals in which secreted or membrane-tethered UNC-6 was overexpressed in AVA neurons (Figure 5D), indicating potentiation of the PHB-mediated response. This is consistent with the observed increase in NLG-1 GRASP signal in wild-type animals with overexpression of the same transgenes. Finally, consistent with *unc-6 *being a limiting factor in *unc-6 *and *unc-40-*mediated SPR, SDS sensitivity is not potentiated in animals overexpressing UNC-40 in PHB neurons (Figure 5D).

## Discussion

Recognition of the correct synaptic partner is an essential final step in neural circuit formation. Using a new approach combining NLG-1 GRASP technology and a circuit-specific behavioral assay, we visualize and test the function of specific synapses in live animals. We have identified the conserved UNC-6/Netrin and UNC-40/DCC ligand-receptor pair as key mediators of SPR between PHB sensory neurons and AVA interneurons. Recognition of the correct synaptic partner is achieved through secretion of limiting amounts of UNC-6 from postsynaptic AVA neurons. UNC-6 signals in a juxtacrine manner to presynaptic PHB neurons via UNC-40/DCC to promote synaptogenesis with individual postsynaptic neurons, despite the presence of over 30 potential partners in the region.

### UNC-6/Netrin and UNC-40/DCC are required for SPR

Conservation of UNC-6/Netrin and UNC-40/DCC functions has been demonstrated for early steps in circuit formation - cell migration, axon guidance, and presynaptic component localization to neuronal compartments. We demonstrate here a novel role for this conserved receptor-ligand pair in the final circuit formation step, namely SPR. A failure to recognize the correct synaptic partner likely has two phenotypic consequences: defects in forming synapses with the correct partners and defects in adhering to the correct partners within a target region. Analysis of PHB-AVA NLG-1 GRASP signals and SDS response in live animals indicates that *unc-6 *and *unc-40 *mutants are defective in their ability to form synapses between PHB and AVA neurons. NLG-1 GRASP defects are not solely explained by defects in NLG-1 transport or expression, as *nlg-1 *mutants respond normally to SDS, consistent with a defect in PHB-AVA synapse formation. In addition, neurite contact is reduced by approximately 25% along the length of neurite overlap.

Localization of presynaptic vesicles to the distal PHB axon is normal in mutants that affect PHB's primary postsynaptic partners, indicating that PHB-AVA SPR is likely downstream of vesicle targeting. Similarly, localization of synaptic components to the distal axon is normal in *unc-40 *and *unc-6 *mutants. This finding contrasts with studies on localization of presynaptic vesicles in AIY interneurons. Thus, in AIY neurons UNC-40 attracts presynaptic components to the middle segment of the neurite, while in PHB neurons presynaptic components are transported from the cell body to the distal region of the axon independently of UNC-40, perhaps by motor proteins implicated in other neurons [43].

Interestingly, although defects in PHB-AVA synaptogenesis are severe in *unc-6 *and *unc-40 *mutants (approximately 50% reduction), some PHB-AVA synaptogenesis persists in the mutant animals. As *unc-6(ev400) *and *unc-40(e271) *are likely null alleles, this indicates that other molecules may function in parallel to the *unc-6-unc-40 *signaling cassette. However, PHB circuit-specific behavior is completely disrupted. The NLG-1 GRASP approach tests the ability to recognize and form synaptic structures with the correct partner, while the circuit-specific behavioral assay tests whether the synapses formed are sufficient for a behavioral response. Thus, the response to SDS may be completely defective because the reduced number of PHB-AVA synapses in *unc-40 *mutants are insufficient for circuit function.

On the other hand, adhesion between synaptic partners is only partially disrupted in *unc-40 *and *unc-6 *mutants. Persisting contact along approximately 73% of the region of neurite overlap may result from molecules that act in parallel to *unc-6 *and *unc-40 *or to other interactions within the nerve bundle. The severe defects in PHB-AVA synaptogenesis are not likely a direct consequence of defects in PHB-AVA neurite contact because *unc-7/Innexin *mutants have PHB-AVA neurite contact defects as severe as *unc-6 *and *unc-40 *mutants, yet display no reduction in synaptogenesis. SPR defects are also unlikely to result from general nerve bundle defasciculation, as fasciculation mutants [32,33] display no defects in NLG-1 GRASP intensity. Consistent with this, in wild-type animals, synapses occupy only 22% of the contact region; thus, 73% contact along the region of neurite overlap should support synaptogenesis. Failure of neurite adhesion and synaptogenesis are thus two separable consequences of the inability to recognize the correct synaptic partner.

### Limiting amounts of UNC-6/Netrin promote synaptogenesis through a signal from postsynaptic to presynaptic neurons

Cell-specific rescue experiments indicate that UNC-6 acts in postsynaptic AVA neurons, and UNC-40 acts in presynaptic PHB neurons to mediate SPR. Furthermore, overexpression of UNC-6 in AVA neurons is sufficient to promote increased PHB to AVA synaptogenesis. Overexpression of UNC-40 in PHB does not promote additional synapses, consistent with a model in which UNC-6 is a limiting signal for regulating synaptogenesis, and unbound UNC-40 cannot instruct SPR. This model is supported by *in vivo *localization data indicating that UNC-6 is present in puncta dispersed in the AVA neurite [42], while UNC-40 is localized along the entire region of PHB that contacts AVA.

Despite the large body of prior work on UNC-6/Netrin and UNC-40/DCC, there have not been any *in vivo *studies reporting function of these molecules in pre- and post-synaptic partners to promote SPR. In fact, during axon guidance, UNC-6/Netrin is frequently secreted from guidepost cells located in or near intermediate or final target regions, but not from specific postsynaptic neurons [4-6]. Similarly, localization of synaptic components in AIY neurons is guided by UNC-6 secretion from a non-neuronal sheath cell [8]. Thus, our results indicate a novel site of UNC-6/Netrin and UNC-40/DCC function, namely in post- and presynaptic neurons, respectively. Signaling between synaptic partners by a Netrin family protein is not without precedent, as *in vitro *experiments indicate that vertebrate GPI-linked Netrins (Netrin Gs) can promote synaptogenesis between partners (reviewed in [45]). However, the underlying molecular mechanisms are likely different since the Netrin Gs are presynaptic rather than postsynaptic, and their binding partners are Netrin G Ligands rather than DCC [12].

### A diffusible molecule mediates a juxtacrine SPR signal

Our results suggest that UNC-6 functions in close proximity to the postsynaptic membrane, because an artificially membrane-tethered UNC-6 expressed in postsynaptic AVAs can promote synapse formation with PHB neurons. The effects are less robust than with secreted UNC-6, which may be due to decreased activity caused by fusion to a heterologous membrane tether. Support for a short-range adhesive capacity for UNC-6/Netrin comes from studies in the developing mammary gland that indicate that Netrin mediates adhesion between preluminal cells expressing Netrin-1 and cap cells expressing the DCC paralog Neogenin [46]. Furthermore, biochemical fractionation studies indicate that a large proportion of Netrin remains membrane-bound in vertebrates [47,48], perhaps by binding to transmembrane receptors or proteins in the extracellular matrix.

**Figure 6:**
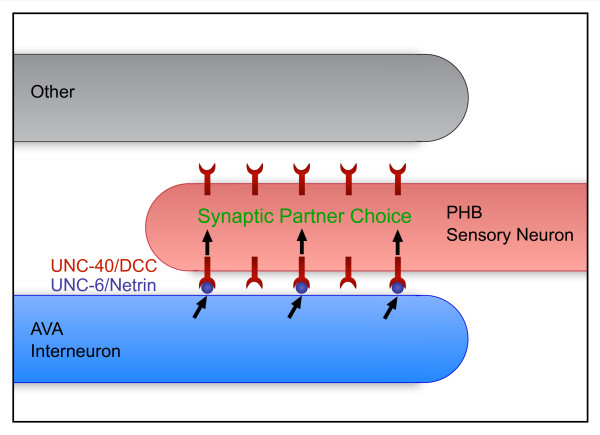
**Limiting amounts of UNC-6/Netrin promote SPR through a juxtacrine signal from postsynaptic to presynaptic neurons**. In this model, UNC-6 secreted from AVA interneurons binds UNC-40 expressed in PHB neurons to direct SPR. Limiting amounts of UNC-6 sequestered near the AVA membrane bind a subset of the available UNC-40 receptors in PHB, inducing a recognition event that results in correct adhesion and synaptogenesis between the two neurons.

We propose that UNC-6 and UNC-40 precisely specify the region of contact between PHB and AVA for synaptogenesis by recruiting presynaptic vesicles in the distal axon to the PHB neurite membrane that abuts AVA (Figure 6). The ability of UNC-40/DCC receptors to recruit presynaptic vesicles is suggested by published studies demonstrating that glia expressing UNC-6 promote recruitment of presynaptic components to specific neurite subdomains via UNC-40 [8]. A restricted, local UNC-6/Netrin signal from an adjacent neurite may allow an axon to distinguish and form synapses with an individual partner among multiple potential neurons.

How is the SPR signal sensed in AVA? One possibility is that a transmembrane receptor expressed in AVA binds UNC-40-bound UNC-6, transducing a signal into AVA and recruiting postsynaptic components. Such a co-receptor would also provide a mechanism for retaining UNC-6/Netrin near the membrane. Expression of UNC-6 and UNC-40 alone is unlikely to promote synaptogenesis with other neurons in the preanal ganglion, since each protein is expressed in additional cells that do not form synapses with either PHB or AVA neurons. This similarly suggests a co-receptor or downstream signal transducer is necessary in each cell. Interestingly, the UNC-6 and UNC-40-mediated SPR signal appears to employ a molecularly distinct pathway from the UNC-6 and UNC-40-mediated cell migration and axon guidance signals, so further investigating this is an interesting avenue for future studies.

### Multiple steps in circuit formation require UNC-6/Netrin and UNC-40/DCC

UNC-6 and UNC-40 regulate three of the four circuit formation steps for PHB neurons, likely through expression of UNC-6 in distinct cell types. At a low frequency in these mutants, the PHB cell body is slightly misplaced within the tail region, indicating defects in cell migration [28] (data not shown). Furthermore, PHB axons often fail to migrate ventrally and enter their target region. (Localization of presynaptic components to the distal axon is normal, indicating that this penultimate step is UNC-6 and UNC-40-independent in PHB.) Finally, SPR within the preanal ganglion is disrupted. Interestingly, we found that UNC-6 secreted by AVA neurons does not rescue axon guidance, consistent with previous studies indicating that ventral PHB axon guidance is directed by UNC-6 secretion from ventral epidermoblasts, PVT, and PVQ [30]. Thus, UNC-6 expression in different cells plays distinct roles in circuit formation. First, expression from an unknown source directs cell migration. Second, expression in ventral epidermoblasts, PVT and PVQ cells guides PHB neurites ventrally to their target region. Finally, local expression in AVA instructs PHB neurons to form synapses with the correct partner within a complex nerve bundle.

Conservation of UNC-6/Netrin and UNC-40/DCC-mediated cell and axon migration signals suggests that the SPR signal may also be conserved in vertebrates. A recent study in *Xenopus *indicates that microinjection of Netrin-1 can transiently increase presynaptic specializations in retinal ganglion cells, and this addition can be blocked by microinjection of anti-DCC antibodies [49]. Furthermore, disruption of DCC function in cultured dopaminergic (DA) neurons reduces the number of autaptic synapses, synapses made by a DA neuron onto itself [50]. However, future studies in vertebrate systems will be needed to determine whether Netrin and DCC mediate direct juxtacrine interactions between pre- and postsynaptic neurons that specify the formation of functional synapses.

### A new approach: combining NLG-1 GRASP and circuit-specific behavior to probe SPR

NLG-1 GRASP presents unique advantages that made our discovery possible. In this study, NLG-1 GRASP was employed to identify genes that mediate the ability of PHB sensory neurons to recognize and form synapses with AVA interneurons within a dense synaptic region. It would have been impossible to uncover a role for *unc-6 *and *unc-40 *in SPR using conventional presynaptic labeling, because earlier steps in circuit formation are unaffected when SPR is disrupted. Another advantage of the NLG-1 GRASP marker is the ability to characterize partially penetrant SPR phenotypes such as those observed in *unc-6 *and *unc-40 *mutants, which require analysis of many animals (approximately 40 per genotype) to describe the population. Similarly, analysis of transgenic rescue and overexpression experiments requires analysis of a statistically significant population of animals (approximately 40 animals from each transgenic line). This is prohibitively time-consuming and labor-intensive with current methods such as electron microscopy reconstruction, which requires months to years for each animal. Importantly, the addition of a circuit-specific behavioral assay allowed us to confirm the function of these synapses in live animals with similarly large sample sizes. Therefore, this new approach allows quantitative genetic analysis of SPR in live animals and shows great promise for discovery and characterization of genes that mediate this critical process in neuronal development.

## Materials and methods

### Strains and genetics

Wild-type strains were *C. elegans *variety Bristol, strain N2. Except for strains containing the *wyEx2309*, *wyEx1098*, *wyEx2871*, *iyEx82*, *iyEx83*, *iyEx84*, *and iyEx85 *transgenes, all strains contain the integrated *_p_nlp-1::mCherry *(10 ng/μl), *_p_flp-18::mCherry *(5 ng/μl), *_p_gpa-6::nlg-1::spGFP1-10 *(60 ng/μl), *_p_flp-18::nlg-1::spGFP11 *(30 ng/μl), and *_p_odr-1::RFP *(20 ng/μl) transgene *wyIs157 *IV. Strains were maintained by standard methods [51]. All worms were raised on OP50 *Escherichia coli-*seeded NGM plates at 20°C.

Mutations and integrated transgenes used in this study include *age-1(hx546) II*, *cfi-1(ky651) I*, *clec-38(tm2035) V*, *ina-1(gm39) III*, *mig-10(ct41) III*, *nlg-1(ok259) X*, *sdn-1(ok449) X*, *unc-5(e53) IV*, *unc-6(ev400) X*, *unc-7(e5) X*, *unc-34(gm104) V*, *unc-40(e271) I*, *unc-42(e419) V*, *unc-104(e1265) II*, *unc-115(ky275) X*, *unc-129(ev554) IV*, and *wyIs157 IV*. Transgenes maintained as extrachromosomal arrays include two lines used for cell-specific rescue of *unc-6(ev400) X*: *iyEx1 *and *iyEx5 *(*_p_rig-3::unc-6 *(40 ng/μl), *_p_unc-122::RFP *(20 ng/μl)), two lines used for cell-specific rescue of *unc-6(ev400) X *with a membrane-tethered *unc-6*: *iyEx19 *and *iyEx21 *(*_p_rig-3::MTunc-6 *(30 ng/μl and 25 ng/μl, respectively), *_p_unc-122:: RFP *(20 ng/μl)), two lines used for cell-specific rescue of *unc-40(e271) I*: *iyEx33 *and *iyEx58 *(*_p_nlp-1::unc-40 *(10 ng/μl), *_p_unc-122:RFP *(20 ng/μl)). Extrachromosomal transgenic arrays used for overexpression experiments include two lines used for overexpression of *unc-6*: *iyEx47 *and *iyEx48 *(*_p_rig-3::unc-6 *(40 ng/μl), *_p_unc-122:RFP *(20 ng/μl)), two lines used for overexpression of membrane-tethered *unc-6: iyEx56 *and *iyEx60 *(*_p_rig-3::MTunc-6 *(30 ng/μl and 25 ng/μl, respectively), *_p_unc-122:RFP *(20 ng/μl)), and three lines used for overexpression of *unc-40*: *iyEx53*, *iyEx52 *and *iyEx54 *(*_p_nlp-1::unc-40 *(5 ng/μl), *_p_unc-122:RFP *(20 ng/μl)). The extrachromosomal transgenic array used for presynaptic or postsynaptic localization in wild type, *unc-6/Netrin*, and *unc-40/DCC *in Figure 3A-F,3I was *wyEx2309 *(*_p_nlp-1::mCherry::rab-3 *(1 ng/μl), *_p_flp-18::nlg-1::YFP *(30 ng/μl), *_p_unc-122::RFP *(30 ng/μl)), in wild type and *cfi-1; unc-42 *in Figure 3G-H,3J was *wyEx1098 *(*_p_nlp-1::mCherry::rab-3 *(10 ng/μl), *_p_odr-1::RFP *(50 ng/μl)), in wild type and *unc-6/Netrin *in Figure S5A-D,S in Additional file 5 was *iyEx84 *(*_p_gpa-6::syd-2::YFP *(30 ng/μl), *_p_unc-122::RFP *(20 ng/μl)), in wild type and *unc-40/DCC *in Figure S5E,F,S in Additional file 5 was *iyEx85 *(*_p_gpa-6::syd-2::YFP *(30 ng/μl), *_p_unc-122::RFP *(20 ng/μl)), in wild type, *unc-6*, and *unc-40 *in Figure S5G-L,S in Additional file 5 was *iyEx83 *(*_p_gpa-6::GFP::elks-1 *(30 ng/μl), *_p_unc-122::RFP *(20 ng/μl)), and in wild type, *unc-6*, and *unc-40 *in Figure S5M-R,S in Additional file 5 was *iyEx82 *(*_p_flp-18::nlg-1::YFP *(30 ng/μl), *_p_nlp-1::mCherry::rab-3 *(0.5 ng/μl), *_p_unc-122::RFP *(20 ng/μl)). The extrachromosomal transgenic array used for *unc-40 *localization in both wild-type and *unc-6 *mutant backgrounds is *wyEx2871 *(*_p_gpa-6::unc-40::GFP *(35 ng/μl), *_p_unc-122::RFP *(20 ng/μl)). Extrachromosomal arrays used for rescue or overexpression were tested in the F4 to F10 generations.

### Cloning and constructs

The following plasmids and transgenic strains were generated using standard techniques: *_p_flp-18::mCherry *[19], *_p_flp-18::nlg-1::spGFP11 *[19], *_p_unc-122::RFP *[52], *_p_odr-1::RFP *[53] were previously described. To generate the *_p_nlp-1::mCherry *construct, the *nlp-1 *promoter was amplified from N2 genomic DNA, adding 5' NotI and 3' XbaI sites, and subcloned into the NotI-XbaI fragment from *_p_ttx-3::mCherry *[8], replacing the *ttx-3 *promoter. To generate the *_p_gpa-6::nlg-1::spGFP1-10 *construct, the *gpa-6 *[24] promoter was amplified from N2 genomic DNA, adding 5' SphI and 3' SmaI sites, and subcloned into SphI-SmaI fragment *nlg-1::spGFP1-10 *[19]. To generate the *_p_rig-3::unc-6 *construct, the SphI-AscI *rig-3 *promoter fragment from *_p_rig-3::CD4-2::spGFP1-10 *[19] was subcloned into the SphI-AscI fragment from *_p_egl-20::unc-6 *[9], replacing the *egl-20 *promoter. To generate the *_p_rig-3::MTunc-6::mCherry *(called *_p_rig-3::MTunc-6 *in the text) construct, the SphI-AscI *rig-3 *promoter fragment was subcloned into the SphI-AscI fragment from *_p_unc-6::MTunc-6::mCherry *(Daniel Colón-Ramos, personal communication), replacing the *unc-6 *promoter. To generate the *_p_nlp-1::unc-40 *construct, site-directed mutagenesis (Stratagene QuikChange Multi Site-Directed Mutagenesis Kit Santa Clara, CA, USA) was used to mutate an internal SphI site in the *nlp-1 *promoter, and the SmaI-SphI *nlp-1 *promoter fragment was subcloned into the SmaI-SphI fragment from *_p_mig-13::unc-40 *(Hannah Teichmann, personal communication), replacing the *mig-13 *promoter. To generate the *_p_gpa-6::unc-40::GFP *construct, the SphI-SmaI *gpa-6 *promoter fragment was subcloned into the SphI-SmaI fragment from *_p_mig-13::unc-40::GFP *(Hannah Teichmann, personal communication), replacing the *mig-13 *promoter. To generate the *_p_nlp-1::mCherry::rab-3 *construct, the NotI-XbaI *nlp-1 *promoter fragment was subcloned into the NotI-XbaI fragment from *_p_ttx-3::mCherry::rab-3 *[8], replacing the *ttx-3 *promoter. To generate the *_p_flp-18::nlg-1::YFP *construct, the SphI-AscI *flp-18 *promoter fragment was subcloned into the SphI-AscI fragment from *_p_opt-3::nlg-1::YFP *[19], replacing the *opt-3 *promoter.

To generate the *_p_gpa-6::syd-2::YFP *construct, *_p_gpa-6::GFP *was first generated by amplifying the *gpa-6 *promoter from N2 genomic DNA, adding 5' NotI and 3' BamHI sites, and subcloning it into the NotI-BamHI fragment of *pSM::GFP*. The NheI-ApaI fragment from *_p_unc-86::syd-2::YFP *[54] was then subcloned into the NheI-ApaI fragment from *_p_gpa-6::GFP*, replacing the *GFP *cDNA. To generate the *_p_gpa-6::GFP::elks-1 *construct, the SphI-SmaI *gpa-6 *promoter fragment was subcloned into the SphI-SmaI fragment from *_p_unc-86::GFP::elks-1 *[54], replacing the *unc-86 *promoter. To generate the *_p_gpa-6::mCherry::rab-3 *construct, the SphI-SmaI *gpa-6 *promoter fragment was subcloned into the SphI-SmaI fragment from *_p_flp-18::mCherry::rab-3 *[19], replacing the *flp-18 *promoter. The *_p_flp-18::nlg-1::YFP *construct was generated using a carboxy-terminal YFP Gateway Destination vector [54] with the *flp-18 *promoter. cDNAs in attL containing pDONR201 vector (from OpenBiosystems Huntsville, AL, USA) encoding NLG-1 was recombined into the carboxy-terminal YFP pSM Gateway Destination vector with LR clonase (Invitrogen Carlsbad, CA, USA).

### Fluorescence microscopy

All images captured were of live *C. elegans *using a Zeiss Axio Imager.A1 compound fluorescent microscope under 630 × magnification. For phenotypic quantification, all micrographs were taken of larval stage animals: L1 to L2 stage animals (Figures 3 and 4E,F; Additional file 5 and Figure S6CC-FF in Additional file 6), L3 stage animals (Figure 2D-L), and L4 stage animals (Figures 1, 2M,N, and 4A-D; Additional files 1, 2, 3, and 4, Figure S6A-BB in Additional file 6 and Additional file 7). Dye-filling of animals with *_p_gpa-6::syd-2::YFP*, *_p_gpa-6::GFP::elks-1*, and *_p_flp-18::nlg-1::YFP *was performed as previously described [55] using 6 μg/ml of DiI to identify and exclude animals with axon guidance defects and to determine the region in which PHB contacts AVA. Animals were immobilized using 0.3 M 2,3-butanedione monoxime (BDM) and 10 mM levamisole in a 2:1 ratio or 10 mM levamisole alone.

### Phenotypic quantification

NIH ImageJ [27] was used to quantify all data from images. This includes PHB-AVA NLG-1 GRASP intensity, *_p_nlp-1::mCherry::rab-3 *intensity, PHB-AVA neurite contact, and NLG-1 GRASP synaptic length. Punctal intensity for NLG-1 GRASP and mCherry::RAB-3 was determined by outlining each cluster of puncta and measuring the intensity at each pixel. To accommodate differences in background fluorescence, background intensity was approximated by determining the minimum intensity value in a region immediately surrounding the puncta. This value was then subtracted from the intensity for each pixel before calculating the sum of the adjusted intensity values. Median intensity values were normalized to wild-type levels measured during the same week on the same microscope. Neurite contact was measured by measuring the length of each gap in contact and the length of PHB-AVA neurite overlap. Percent contact was calculated by subtracting the sum of the gap lengths from the length of overlap, and dividing by the length of overlap in each animal. The synaptic length as a proportion of length of neurite overlap was calculated by measuring the length (along the anterior-posterior axis) of each NLG-1 GRASP punctum or cluster of puncta, and summing these lengths. Percent synaptic length was calculated by subtracting the sum of the synaptic lengths from the length of overlap, and dividing by the length of overlap in each animal. The percent animals with normal axon guidance was determined by adding the total number of animals that have both PHB axons extending into the ventral nerve cord (rather than along a lateral or other tract) and dividing it by the total number of animals assayed. The percentage of gaps in each region was quantified by tallying the number of gaps between parallel neurites in the anterior, medial, and posterior regions of the preanal ganglion and dividing by the total number of gaps.

### SDS-avoidance behavior

A nose touch behavior assay was developed to test PHB-AVA function by adapting a published dry drop test [44]. An adult was placed on a dry, unseeded NGM plate. A hair pick was then used to touch the nose of the animal to stimulate ASH-AVA sensory neuron to direct backward movement. Once the animal began backing, a drop of M13 buffer or repellent (M13 buffer with 0.1% SDS) was placed on the agar near the tail of the moving worm using a mouth pipette. As soon as the solution contacts the agar, the drop is absorbed and the animal backs into the dry drop solution. A stopwatch was used to record the amount of time that the animal backs into the drop before stopping.

We tested the response of at least 40 adults to the control M13 buffer and at least 40 adults to 0.1% SDS (in M13) to find the individual backing times for both wild-type and mutant or transgenic animals. In strains with PHB or AVA axon guidance defects, animals with wild-type axon guidance were isolated using the Axio Imager. A1 compound fluorescent microscope at the L4 stage; anesthetics cause impaired movement and so no anesthetic was used. The behavior assay was performed on these animals at the gravid adult stage the following day. The relative response index was calculated by dividing the average backing time into SDS by the average backing time into buffer to account for slow movement in mutants or transgenic lines. This calculated value is divided by the same value for wild-type animals to normalize the wild-type response index to 100%.

### Statistical analysis

Median values for relative intensity were compared first by a Kruskal-Wallis test, a nonparametric alternative to ANOVA that does not rely on a normality assumption. It tests whether the medians (rather than means) of k independent groups are all equal. If the result is found to be non-significant (*P *> 0.05), it is not necessary to follow up with pair-wise comparisons. If the result of the Kruskal-Wallis test is found to be significant, which means that the medians of at least two of the groups differ significantly, it is followed up by a Mann-Whitney u-test, which compares the medians of the two independent groups. If more than one u-test is conducted, the resulting *P*-values are adjusted for multiple comparisons by the Hochberg method. The Hochberg procedure is a standard procedure applied to adjust for the tendency to incorrectly reject a null hypothesis when multiple comparisons are made, and can only conservatively increase *P*-values. A multiple comparison procedure is required whenever several conclusions are drawn from the same group of data in order to assure that the overall error rate for a type I error is still bound by the chosen significance level (here 0.05, 0.01, or 0.001).

Results are reported in the form of *P*-values in figures (*P < 0.05, ** *P *< 0.01, *** *P *< 0.001, NS *P *> 0.05) and exact *P*-values are given in Additional file 10 rather than error bars. *P*-values provide precise information about whether two samples differ significantly in either means or medians. The procedure by which *P*-values are obtained incorporates information on the sample sizes and variability in both samples, making it unnecessary for the reader to extrapolate this information from error bars, which would require multiplying each standard error of the mean by a factor that depends on each sample size.

Data representing mean length or means of percentages were analyzed by ANOVA, and if significant, with pair-wise *t*-tests followed by the Hochberg procedure for multiple comparisons. For comparing relative SDS response indices, in addition to the *t*-tests described in Figure 5, a multi-way ANOVA model with appropriate interaction terms was fitted using the linear model procedure in R statistical computing software [56] and ANOVA *post hoc *tests were performed to confirm statistical significance (Additional file 10).

## Abbreviations

DCC: Deleted in colorectal cancer; GFP: green fluorescent protein; GPI: glycosyl phosphatidylinositol; GRASP: GFP reconstitution across synaptic partners; IgSF: immunoglobulin superfamily; NLG: Neuroligin; SDS: sodium dodecyl sulfate; SPR: synaptic partner recognition; YFP: yellow fluorescent protein.

## Competing interests

The authors declare that they have no competing interests.

## Authors' contributions

JP, PLK, and MKV conceived and designed the experiments. JP, PLK, WW, SNO, AG, KLB, BJB, MR, NM, and MKV performed the experiments. JP, PLK, KLB, BJB, MB, and MKV analyzed the data. JP, WW, MB and MKV wrote the paper. All authors read and approved the final manuscript.

## Supplementary Material

Additional file 1 **Figure S1 - gaps in neurite contact show no preference for a particular region**. Posterior, medial, and anterior gaps correspond to one-third the region of overlap between PHB and AVA. Animals with gaps in more than one region were counted for each category. A χ^2 ^goodness-of-fit test was performed to compare observed ratios to 33.3%, which is expected by chance for the three regions; *P *> 0.05. For percent gaps in each region, *unc-6 *n = 42 animals and *unc-40 *n = 40 animals. Click here for file

Additional file 2 **Figure S2 - mutants that disrupt neurite contact and fasciculation exhibit no defects in PHB-AVA synaptogenesis**. **(A,B) **Quantification of NLG-1 GRASP intensity (A) and neurite contact (B) for *unc-7*, *ina-1*, and *sdn-1 *mutants. (A) *unc-7*, *ina-1*, and *sdn-1 *have normal NLG-1 GRASP intensities, indicating that PHB-AVA synaptogenesis is not affected by the observed defects in PHB-AVA neurite contact or general nerve bundle fasciculation. Wild-type n = 216, *unc-6 *n = 87, *unc-40 *n = 85, *unc-7 *n = 39, *ina-1 *n = 41, and *sdn-1 *n = 41 animals. ***P < 0.001, NS, not significant, u-test. *P*-values were adjusted for multiple comparisons using the Hochberg method. (B) *unc-7 *mutants display defects in PHB-AVA neurite contact as severe as those in *unc-6 *and *unc-40 *mutants. *ina-1 *mutants display less severe neurite contact defects, and *sdn-1 *mutants do not have significant defects in neurite contact, although both mutants have previously characterized defects in general nerve bundle fasciculation. Wild-type n = 216, *unc-6 *n = 87, *unc-40 *n = 85, *unc-7 *n = 39, *ina-1 *n = 41, and *sdn-1 *n = 41 animals. ***P < 0.001, **P < 0.01, NS, not significant, *t*-test. *P*-values were adjusted for multiple comparisons using the Hochberg method. **(C,G,K,O) **Schematics, **(D,H,L,P) **micrographs of PHB-AVA NLG-1 GRASP signal, **(E,I,M,Q) **cytosolic mCherry labeling PHB and AVA neurite contact, and **(F,J,N,R) **merged images. (C-F) Wild type, (G-J) *unc-7*, (K-N) *ina-1*, (O-R) *sdn-1*. Yellow scale bar: 2 μm. Click here for file

Additional file 3 **Figure S3 - in wild-type animals synapses occupy a fraction of the length of neurite contact between PHB and AVA**. Quantification of the extent of PHB-AVA neurite overlap, and the sum of the lengths of all NLG-1 GRASP puncta in the preanal ganglion in wild-type animals. The sum of the lengths of all NLG-1 GRASP puncta indicates that only 22% of the neurite overlap region is occupied by PHB-AVA synapses. Wild-type n = 41 animals. ***P < 0.001, *t*-test. *P*-values were adjusted for multiple comparisons using the Hochberg method. Click here for file

Additional file 4 **Figure S4 - *unc-6 *and *unc-40*-mediated cell migration and axon guidance pathway mutants exhibit no defects in SPR**. Quantification of **(A) **NLG-1 GRASP intensity and **(B) **neurite contact for *age-1*, *clec-38*, *mig-10*, *unc-5*, *unc-115*, and *unc-129 *mutants. (A) Axon guidance molecules *age-1*, *clec-38*, *mig-10*, *unc-5*, *unc-115*, *and unc-129 *have normal NLG-1 GRASP intensities, indicating that SPR is molecularly distinct from the classical *unc-6 *and *unc-40*-mediated axon guidance pathways. Wild-type n = 80- 127, *age-1 *n = 131, *clec-38 *n = 91, *mig-10 *n = 99, *unc-5 *n = 80, *unc-115 *n = 86, *unc-129 *n = 88 animals. NS, not significant, u-test. *P*-values were adjusted for multiple comparisons using the Hochberg method. (B) Axon guidance molecules *age-1*, *clec-38*, *mig-10*, *unc-5*, *unc-115*, *and unc-129 *have normal neurite contact, also indicating that the SPR pathway is molecularly distinct. Wild-type n = 80127, *age-1 *n = 131, *clec-38 *n = 91, *mig-10 *n = 99, *unc-5 *n = 80, *unc-115 *n = 86, *unc-129 *n = 88 animals. NS, not significant, *t*-test. *P*-values were adjusted for multiple comparisons using the Hochberg method. **(C,G,K,O,S,W,AA,EE) **Schematics, **(D,H,L,P,T,X,BB,FF) **micrographs of normal PHB-AVA NLG-1 GRASP signal, **(E,I,M,Q,U,Y,CC,GG) **cytosolic mCherry labeling normal PHB and AVA neurite contact, and **(F,J,N,R,V,Z,DD,HH) **merged images. (C-F) Wild type, (G-J) *age-1*, (K-N) *clec-38 *(O-R) *mig-10*, (S-V) *unc-5*, (W-Z) *unc-115*, and (AA-DD) *unc-129 *animal. (EE-HH) *unc-34 *animals could not be assayed for SPR phenotypes due to penetrant neurite extension defects in PHB and sometimes in AVA. Yellow scale bar: 2 μm. Click here for file

Additional file 5 **Figure S5 - active zone and postsynaptic components localize to the correct subcellular compartment in *unc-6 *and *unc-40 *mutants**. **(A,B,G,H,M,N) **Wild type, **(C,D,I,J,O,P) ***unc-6*, and **(E,F,K,L,Q,R) ***unc-40 *are labeled with the active zone markers *syd-2::YFP *(A-F) or *GFP::elks-1 *(G-L) expressed in PHB neurons or the postsynaptic marker *nlg-1::YFP *(M-R) in AVA. (A-L) Presynaptic active zone components are unaltered in *unc-6 *and *unc-40 *mutant animals, localizing to the distal region of the PHB axon within the preanal ganglion where PHB synapses normally form (boxed in white). Yellow scale bar: 2 μm. (M-R) Postsynaptic specializations also localize normally to the preanal ganglion (boxed in white) as well as along the ventral nerve cord where AVA receives synaptic input from other neurons. Yellow scale bar: 2 μm. **(S) **Quantification of pre- or postsynaptic marker fluorescence intensity using NIH ImageJ indicates no significant difference among wild type, *unc-6*, and *unc-40 *animals. For *syd-2::YFP*, wild type n = 3942, *unc-6 *n = 30, and *unc-40 *n = 29 animals. For *GFP::elks-1*, wild type n = 46, *unc-6 *n = 31, *unc-40 *n = 41. For *nlg-1::YFP*, wild type n = 40 to 42, *unc-6 *n = 36, *unc-40 *n = 43. NS, not significant, u-test. *P*-values were adjusted for multiple comparisons using the Hochberg method. Click here for file

Additional file 6 **Figure S6 - expression of UNC-6 in AVA and UNC-40 in PHB are sufficient for SPR**. **(A,E,I,M,Q,U,Y) **Schematics and **(B,F,J,N,R,V,Z) **micrographs of PHB-AVA NLG-1 GRASP signal, **(C,G,K,O,S,W,AA) **cytosolic mCherry labeling PHB and AVA neurite contact, and **(D,H,L,P,T,X,BB) **merged images. (A-D) Wild-type animal, (E-H) *unc-6; _p_AVA::unc-6 *animal, (I-L) *unc-6; _p_AVA::MTunc-6 *animal, (M-P) *unc-40; _p_PHB::unc-40 *animal, (Q-T) animal overexpressing *_p_AVA::unc-6*, (U-X) animal overexpressing *_p_AVA::MTunc-6*, (Y-BB) animal overexpressing *_p_PHB::unc-40*. (A-BB) Yellow scale bar: 2 μm. **(CC) **Schematic and **(DD) **micrograph of *_p_PHB::unc-40::GFP *in wild-type background. **(EE) **Schematic and **(FF) **micrograph of *unc-6; _p_PHB::unc-40::GFP*, indicating that localization of UNC-40 protein to the synaptic region of the PHB axon is not altered in the absence of its ligand UNC-6. (CC-FF) Yellow scale bar: 5 μm. Click here for file

Additional file 7 **Figure S7 - *unc-104 *mutants have similar SPR defects to *unc-6 *mutants**. Quantification of **(A) **NLG-1 GRASP intensity and **(B) **neurite contact in *unc-104 *mutants, which is required for UNC-6 localization. (A) *unc-104 *mutants have defects in NLG-1 GRASP intensity that are similar to those observed in *unc-6 *and *unc-40 *mutants. ***P < 0.001, NS, not significant, u-test. *P*-values were adjusted for multiple comparisons using the Hochberg method. (B) Defects in neurite contact are also similar in *unc-104*, *unc-6*, and *unc-40 *mutants. ***P < 0.001, NS, not significant, *t*-test. *P*-values were adjusted for multiple comparisons using the Hochberg method. (A,B) Wild-type n = 134, *unc-6 *n = 87, *unc-40 *n = 85, *unc-104 *n = 43 animals. Click here for file

Additional file 8 ****Movie S1 - response of a wild-type animal to SDS****. A wild-type animal is touched on the nose with a hair pick, eliciting backward motion. A dry drop of SDS is placed behind the animal. The animal quickly stops reversing when it encounters the SDS. The dry drop of SDS is outlined in red. Click here for file

Additional file 9 **Movie S2 - response of an *unc-40 *animal to SDS**. An *unc-40 *mutant animal is touched on the nose with a hair pick, eliciting backward motion. A dry drop of SDS is placed behind the animal. The response time to SDS is longer than that displayed by wild-type animals. The dry drop of SDS is outlined in red. Click here for file

Additional file 10 **Table S1 - summary of statistical analysis**. *P*-values for all statistical tests performed in this work. Click here for file
