# Supplementary figures and images for: A conserved juxtacrine signal regulates synaptic partner recognition in Caenorhabditis elegans
Source: Neural Dev. 2011 Jun 10;6:28. doi: 10.1186/1749-8104-6-28 (PMC3130637; doi:10.1186/1749-8104-6-28)

## Additional file 1, Figure S1

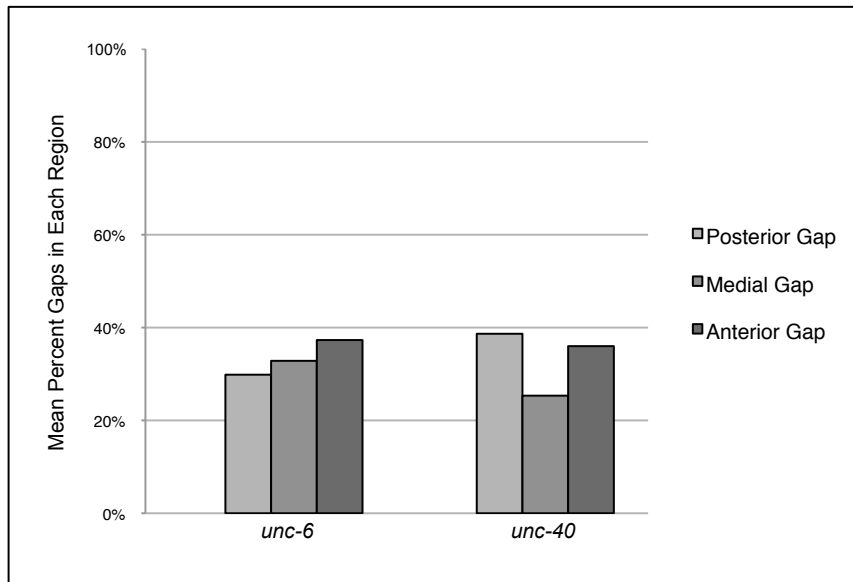

Supplement: Additional file 1 — Figure S1 - gaps in neurite contact show no preference for a particular region. Posterior, medial, and anterior gaps correspond to one-third the region of overlap between PHB and AVA. Animals with gaps in more than one region were counted for each category. A χ2 goodness-of-fit test was performed to compare observed ratios to 33.3%, which is expected by chance for the three regions; P > 0.05. For percent gaps in each region, unc-6 n = 42 animals and unc-40 n = 40 animals. [file 1749-8104-6-28-S1.PDF]

Additional file 2, Figure S2

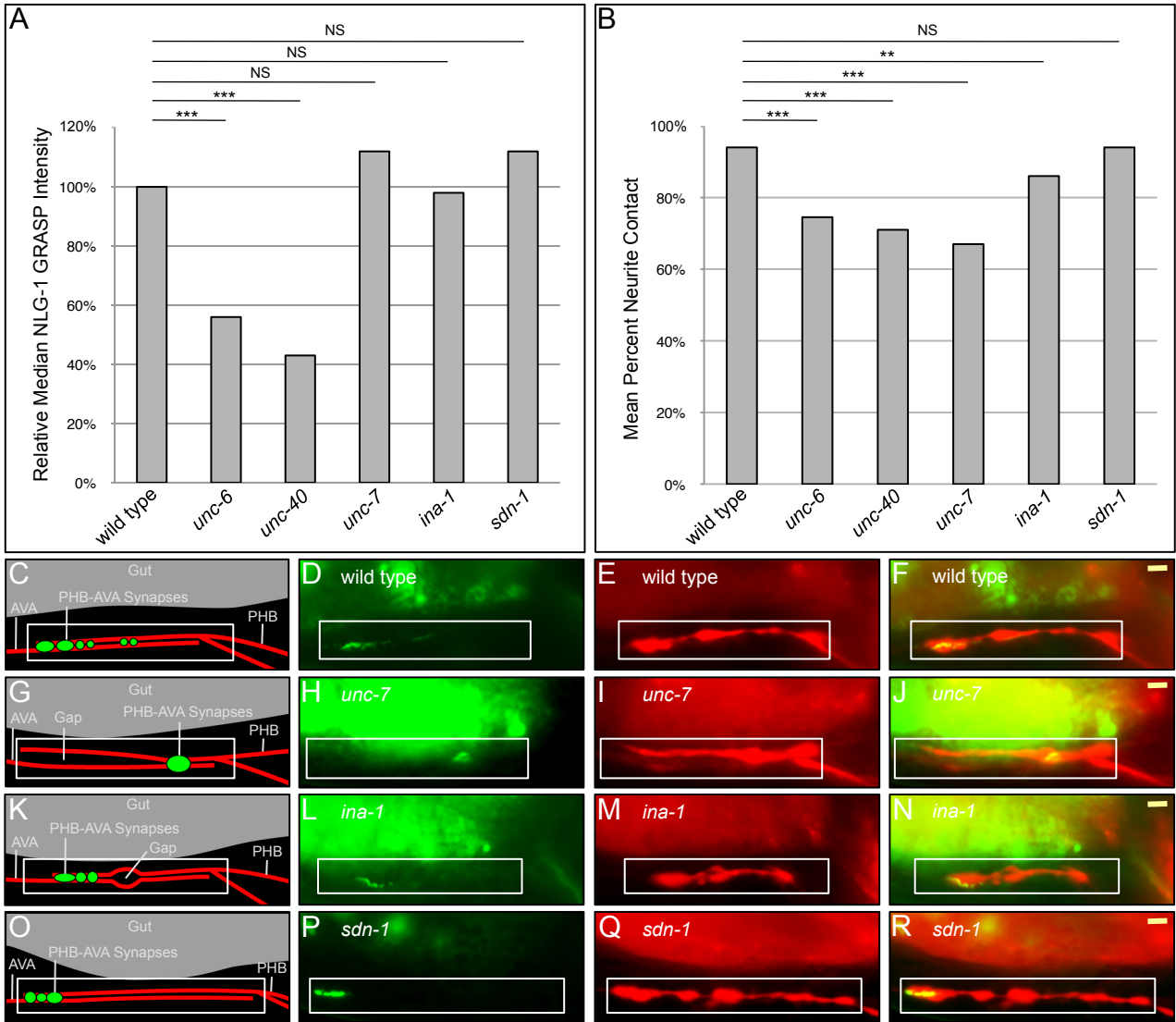

Supplement: Additional file 2 — Figure S2 - mutants that disrupt neurite contact and fasciculation exhibit no defects in PHB-AVA synaptogenesis. (A,B) Quantification of NLG-1 GRASP intensity (A) and neurite contact (B) for unc-7, ina-1, and sdn-1 mutants. (A) unc-7, ina-1, and sdn-1 have normal NLG-1 GRASP intensities, indicating that PHB-AVA synaptogenesis is not affected by the observed defects in PHB-AVA neurite contact or general nerve bundle fasciculation. Wild-type n = 216, unc-6 n = 87, unc-40 n = 85, unc-7 n = 39, ina-1 n = 41, and sdn-1 n = 41 animals. ***P < 0.001, NS, not significant, u-test. P-values were adjusted for multiple comparisons using the Hochberg method. (B) unc-7 mutants display defects in PHB-AVA neurite contact as severe as those in unc-6 and unc-40 mutants. ina-1 mutants display less severe neurite contact defects, and sdn-1 mutants do not have significant defects in neurite contact, although both mutants have previously characterized defects in general nerve bundle fasciculation. Wild-type n = 216, unc-6 n = 87, unc-40 n = 85, unc-7 n = 39, ina-1 n = 41, and sdn-1 n = 41 animals. ***P < 0.001, **P < 0.01, NS, not significant, t-test. P-values were adjusted for multiple comparisons using the Hochberg method. (C,G,K,O) Schematics, (D,H,L,P) micrographs of PHB-AVA NLG-1 GRASP signal, (E,I,M,Q) cytosolic mCherry labeling PHB and AVA neurite contact, and (F,J,N,R) merged images. (C-F) Wild type, (G-J) unc-7, (K-N) ina-1, (O-R) sdn-1. Yellow scale bar: 2 μm. [file 1749-8104-6-28-S2.PDF]

**Additional file 3, Figure S3**

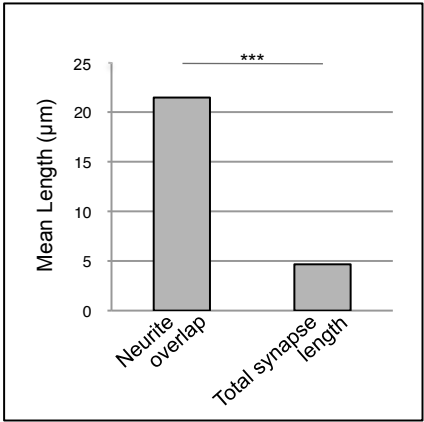

Supplement: Additional file 3 — Figure S3 - in wild-type animals synapses occupy a fraction of the length of neurite contact between PHB and AVA. Quantification of the extent of PHB-AVA neurite overlap, and the sum of the lengths of all NLG-1 GRASP puncta in the preanal ganglion in wild-type animals. The sum of the lengths of all NLG-1 GRASP puncta indicates that only 22% of the neurite overlap region is occupied by PHB-AVA synapses. Wild-type n = 41 animals. ***P < 0.001, t-test. P-values were adjusted for multiple comparisons using the Hochberg method. [file 1749-8104-6-28-S3.PDF]

Additional file 4, Figure S4

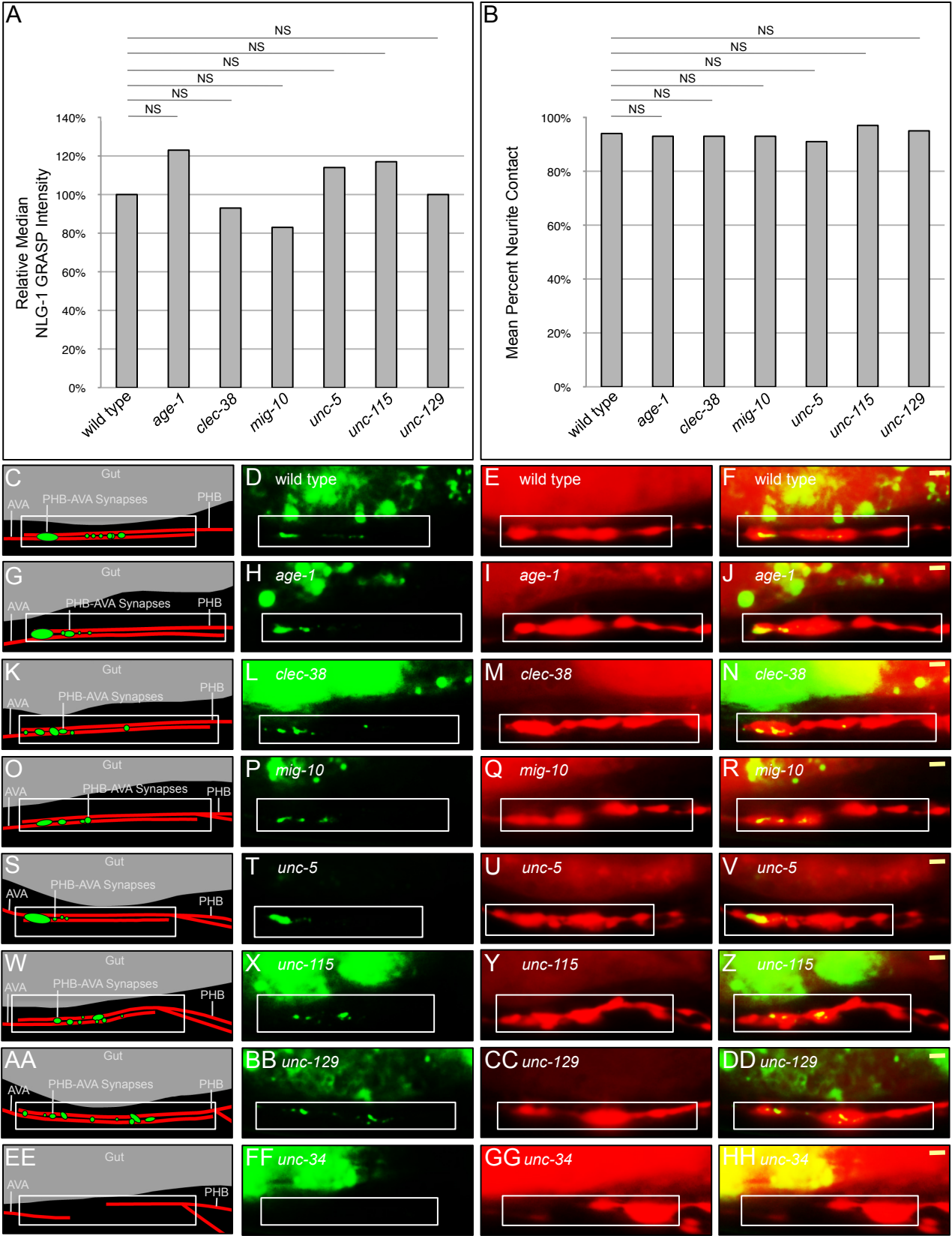

Supplement: Additional file 4 — Figure S4 - unc-6 and unc-40-mediated cell migration and axon guidance pathway mutants exhibit no defects in SPR. Quantification of (A) NLG-1 GRASP intensity and (B) neurite contact for age-1, clec-38, mig-10, unc-5, unc-115, and unc-129 mutants. (A) Axon guidance molecules age-1, clec-38, mig-10, unc-5, unc-115, and unc-129 have normal NLG-1 GRASP intensities, indicating that SPR is molecularly distinct from the classical unc-6 and unc-40-mediated axon guidance pathways. Wild-type n = 80- 127, age-1 n = 131, clec-38 n = 91, mig-10 n = 99, unc-5 n = 80, unc-115 n = 86, unc-129 n = 88 animals. NS, not significant, u-test. P-values were adjusted for multiple comparisons using the Hochberg method. (B) Axon guidance molecules age-1, clec-38, mig-10, unc-5, unc-115, and unc-129 have normal neurite contact, also indicating that the SPR pathway is molecularly distinct. Wild-type n = 80127, age-1 n = 131, clec-38 n = 91, mig-10 n = 99, unc-5 n = 80, unc-115 n = 86, unc-129 n = 88 animals. NS, not significant, t-test. P-values were adjusted for multiple comparisons using the Hochberg method. (C,G,K,O,S,W,AA,EE) Schematics, (D,H,L,P,T,X,BB,FF) micrographs of normal PHB-AVA NLG-1 GRASP signal, (E,I,M,Q,U,Y,CC,GG) cytosolic mCherry labeling normal PHB and AVA neurite contact, and (F,J,N,R,V,Z,DD,HH) merged images. (C-F) Wild type, (G-J) age-1, (K-N) clec-38 (O-R) mig-10, (S-V) unc-5, (W-Z) unc-115, and (AA-DD) unc-129 animal. (EE-HH) unc-34 animals could not be assayed for SPR phenotypes due to penetrant neurite extension defects in PHB and sometimes in AVA. Yellow scale bar: 2 μm. [file 1749-8104-6-28-S4.PDF]

**Additional file 5, Figure S5**

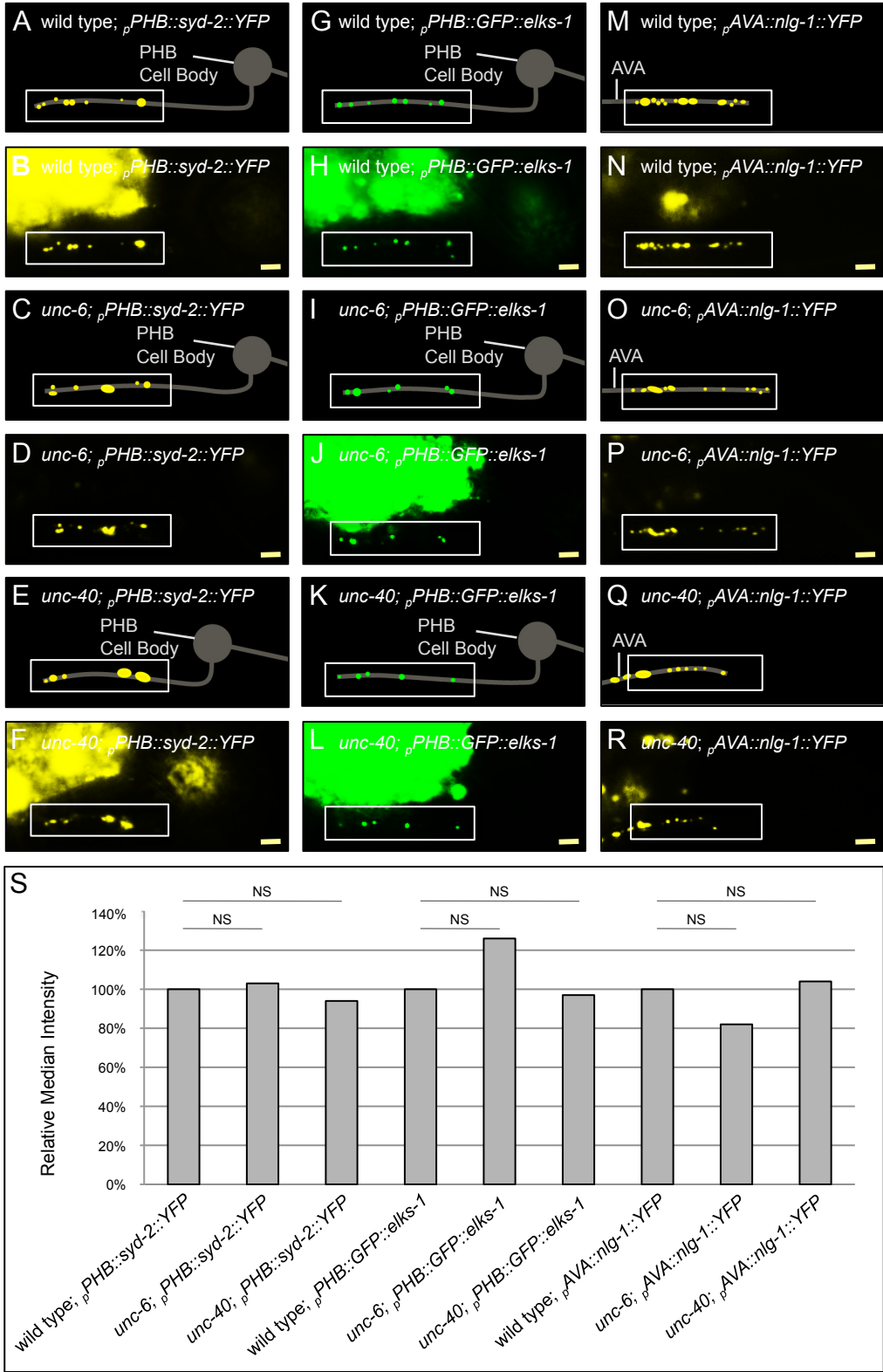

Supplement: Additional file 5 — Figure S5 - active zone and postsynaptic components localize to the correct subcellular compartment in unc-6 and unc-40 mutants. (A,B,G,H,M,N) Wild type, (C,D,I,J,O,P) unc-6, and (E,F,K,L,Q,R) unc-40 are labeled with the active zone markers syd-2::YFP (A-F) or GFP::elks-1 (G-L) expressed in PHB neurons or the postsynaptic marker nlg-1::YFP (M-R) in AVA. (A-L) Presynaptic active zone components are unaltered in unc-6 and unc-40 mutant animals, localizing to the distal region of the PHB axon within the preanal ganglion where PHB synapses normally form (boxed in white). Yellow scale bar: 2 μm. (M-R) Postsynaptic specializations also localize normally to the preanal ganglion (boxed in white) as well as along the ventral nerve cord where AVA receives synaptic input from other neurons. Yellow scale bar: 2 μm. (S) Quantification of pre- or postsynaptic marker fluorescence intensity using NIH ImageJ indicates no significant difference among wild type, unc-6, and unc-40 animals. For syd-2::YFP, wild type n = 3942, unc-6 n = 30, and unc-40 n = 29 animals. For GFP::elks-1, wild type n = 46, unc-6 n = 31, unc-40 n = 41. For nlg-1::YFP, wild type n = 40 to 42, unc-6 n = 36, unc-40 n = 43. NS, not significant, u-test. P-values were adjusted for multiple comparisons using the Hochberg method. [file 1749-8104-6-28-S5.PDF]

**Additional file 6, Figure S6**

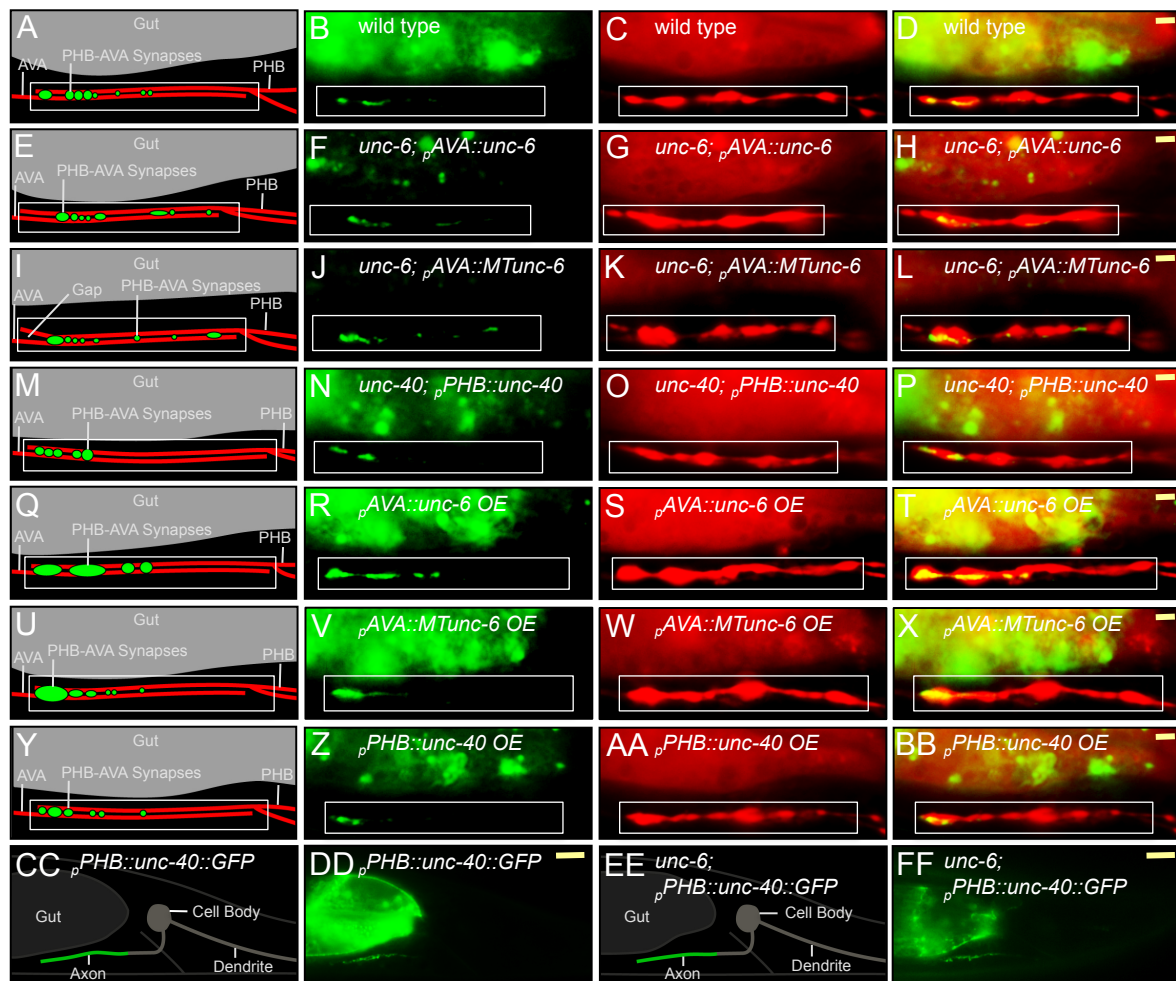

Supplement: Additional file 6 — Figure S6 - expression of UNC-6 in AVA and UNC-40 in PHB are sufficient for SPR. (A,E,I,M,Q,U,Y) Schematics and (B,F,J,N,R,V,Z) micrographs of PHB-AVA NLG-1 GRASP signal, (C,G,K,O,S,W,AA) cytosolic mCherry labeling PHB and AVA neurite contact, and (D,H,L,P,T,X,BB) merged images. (A-D) Wild-type animal, (E-H) unc-6; pAVA::unc-6 animal, (I-L) unc-6; pAVA::MTunc-6 animal, (M-P) unc-40; pPHB::unc-40 animal, (Q-T) animal overexpressing pAVA::unc-6, (U-X) animal overexpressing pAVA::MTunc-6, (Y-BB) animal overexpressing pPHB::unc-40. (A-BB) Yellow scale bar: 2 μm. (CC) Schematic and (DD) micrograph of pPHB::unc-40::GFP in wild-type background. (EE) Schematic and (FF) micrograph of unc-6; pPHB::unc-40::GFP, indicating that localization of UNC-40 protein to the synaptic region of the PHB axon is not altered in the absence of its ligand UNC-6. (CC-FF) Yellow scale bar: 5 μm. [file 1749-8104-6-28-S6.PDF]

Additional file 7, Figure S7

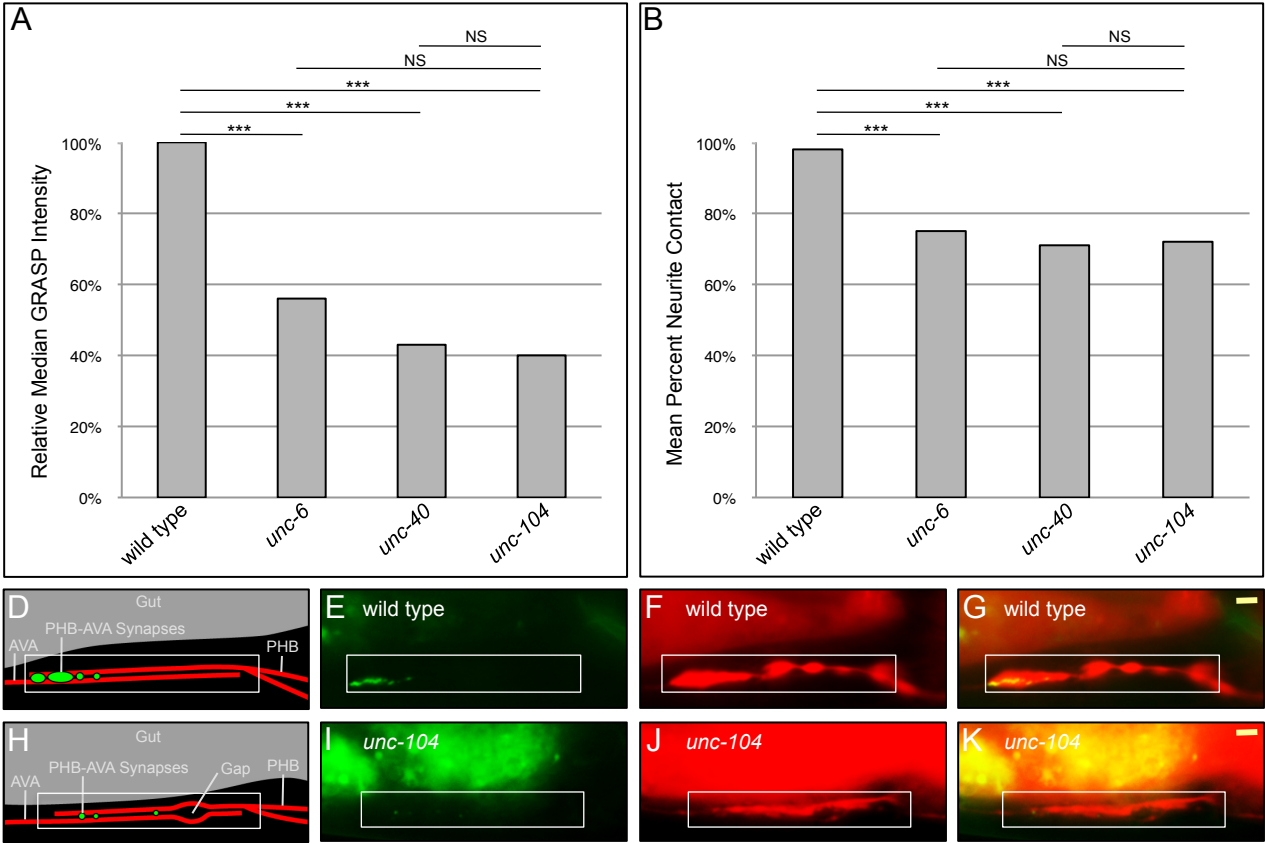

Supplement: Additional file 7 — Figure S7 - unc-104 mutants have similar SPR defects to unc-6 mutants. Quantification of (A) NLG-1 GRASP intensity and (B) neurite contact in unc-104 mutants, which is required for UNC-6 localization. (A) unc-104 mutants have defects in NLG-1 GRASP intensity that are similar to those observed in unc-6 and unc-40 mutants. ***P < 0.001, NS, not significant, u-test. P-values were adjusted for multiple comparisons using the Hochberg method. (B) Defects in neurite contact are also similar in unc-104, unc-6, and unc-40 mutants. ***P < 0.001, NS, not significant, t-test. P-values were adjusted for multiple comparisons using the Hochberg method. (A,B) Wild-type n = 134, unc-6 n = 87, unc-40 n = 85, unc-104 n = 43 animals. [file 1749-8104-6-28-S7.PDF]
