# Supplementary material for: A conserved juxtacrine signal regulates synaptic partner recognition in Caenorhabditis elegans
Source: Neural Dev. 2011 Jun 10;6:28. doi: 10.1186/1749-8104-6-28 (PMC3130637; doi:10.1186/1749-8104-6-28)
Supplement: Additional file 10 — Table S1 - summary of statistical analysis. P-values for all statistical tests performed in this work. [file 1749-8104-6-28-S10.PDF]

Additional Table 1, Table S1

Figure Number	Figure Letter	Genotypes Compared	Statistical Test	Non-Hochberg Adjusted p-value	Hochberg Adjusted p-value
2	M	wild type and *unc-6*	u-test		1.1E-05
2	M	wild type and *unc-40*	u-test		1.1E-07
2	M	*unc-6* and *unc-40*	u-test		5.8E-01
2	N	wild type and *unc-6*	t-test		2.4E-18
2	N	wild type and *unc-40*	t-test		2.4E-18
2	N	*unc-6* and *unc-40*	t-test		2.5E-01
3	I	wild type; *mCherry::rab-3*, *unc-6; mCherry::rab-3*, and *unc-40; mCherry::rab-3*	Kruskal Wallis	5.5E-02	
3	J	wild type; *mCherry::rab-3* and *cfi-1; unc-42; mCherry::rab-3*	u-test	1.2E-01	
4	A	*unc-6* and *unc-6;* $_p$*AVA::unc-6*	u-test		5.0E-03
4	A	*unc-6* and *unc-6;* $_p$*AVA::MTunc-6*	u-test		5.0E-03
4	A	*unc-40* and *unc-40;* $_p$*PHB::unc-40*	u-test		3.2E-07
4	B	*unc-6* and *unc-6;* $_p$*AVA::unc-6*	t-test		3.5E-07
4	B	*unc-6* and *unc-6;* $_p$*AVA::MTunc-6*	t-test		8.3E-03
4	B	*unc-40* and *unc-40;* $_p$*PHB::unc-40*	t-test		9.5E-15
4	C	*unc-6* and *unc-6;* $_p$*AVA::unc-6*	χ^2 goodness-of-fit test		9.5E-01
4	C	*unc-40* and *unc-40;* $_p$*PHB::unc-40*	χ^2 goodness-of-fit test		2.5E-03
4	D	wild type and $_p$*AVA::unc-6 OE*	u-test		4.7E-05
4	D	wild type and $_p$*AVA::MTunc-6 OE*	u-test		3.7E-03
4	D	wild type and $_p$*PHB::unc-40 OE*	u-test		4.8E-01
5	C	wild type (no NLG-1 GRASP) and wild type	t-test		9.1E-01
			Multi-way ANOVA		8.8E-01
5	C	wild type and *nlg-1*	t-test		8.3E-01
			Multi-way ANOVA		8.8E-01
5	C	wild type and *unc-40*	t-test		4.5E-14
			Multi-way ANOVA		8.0E-16
5	C	wild type and *unc-40;* $_p$*PHB::unc-40*	t-test		8.5E-02
			Multi-way ANOVA		6.9E-01
5	C	*unc-40* and *unc-40;* $_p$*PHB::unc-40*	t-test		1.1E-12
			Multi-way ANOVA		8.0E-16
5	D	wild type and $_p$*AVA::unc-6 OE*	t-test		9.9E-18
			Multi-way ANOVA		1.3E-13
5	D	wild type and $_p$*AVA::MTunc-6 OE*	t-test		1.8E-03
			Multi-way ANOVA		2.9E-02
5	D	wild type and $_p$*PHB::unc-40 OE*	t-test		1.8E-01
			Multi-way ANOVA		1.5E-01
S1	A	*unc-6* anterior, medial, and posterior gaps	χ^2 goodness-of-fit test	7.5E-01	
S1	A	*unc-40* anterior, medial, and posterior gaps	χ^2 goodness-of-fit test	3.3E-01	
S2	A	wild type and *unc-6*	u-test		2.3E-05
S2	A	wild type and *unc-40*	u-test		1.8E-07
S2	A	wild type and *unc-7*	u-test		9.4E-01
S2	A	wild type and *ina-1*	u-test		9.4E-01
S2	A	wild type and *sdn-1*	u-test		9.4E-01
S2	B	wild type and *unc-6*	t-test		4.4E-13
S2	B	wild type and *unc-40*	t-test		1.2E-13
S2	B	wild type and *unc-7*	t-test		1.5E-07
S2	B	wild type and *ina-1*	t-test		1.3E-03
S2	B	wild type and *sdn-1*	t-test		9.9E-01
S3	A	wild type neurite overlap and total synapse length	t-test	3.5E-34	
S4	A	wild type and *age-1*	u-test		1.1E-01
S4	A	wild type and *clec-38*	u-test		5.5E-01
S4	A	wild type and *mig-10*	u-test		4.9E-01
S4	A	wild type and *unc-5*	u-test		5.5E-01
S4	A	wild type and *unc-115*	u-test		5.5E-01
S4	A	wild type and *unc-129*	u-test		5.5E-01
S4	B	wild type and *age-1*	t-test		7.1E-01
S4	B	wild type and *clec-38*	t-test		7.1E-01
S4	B	wild type and *mig-10*	t-test		7.1E-01
S4	B	wild type and *unc-5*	t-test		7.1E-01
S4	B	wild type and *unc-115*	t-test		7.1E-01
S4	B	wild type and *unc-129*	t-test		7.1E-01
S5	S	wild type; *syd-2::YFP* and *unc-6; syd-2::YFP*	u-test		9.8E-01
S5	S	wild type; *syd-2::YFP* and *unc-40; syd-2::YFP*	u-test		9.8E-01
S5	S	wild type; GFP::*elks-1* and *unc-6; GFP::elks-1*	u-test		4.6E-01
S5	S	wild type; GFP::*elks-1* and *unc-40; GFP::elks-1*	u-test		7.8E-01
S5	S	wild type; *nlg-1::YFP* and *unc-6; nlg-1::YFP*	u-test		9.8E-01
S5	S	wild type; *nlg-1::YFP* and *unc-40; nlg-1::YFP*	u-test		9.8E-01
S7	A	wild type and *unc-6*	u-test		1.7E-05
S7	A	wild type and *unc-40*	u-test		1.8E-07
S7	A	wild type and *unc-104*	u-test		5.1E-06
S7	A	*unc-6* and *unc-104*	u-test		9.2E-02
S7	A	*unc-40* and *unc-104*	u-test		1.3E-01
S7	B	wild type and *unc-6*	t-test		1.1E-07
S7	B	wild type and *unc-40*	t-test		2.3E-06
S7	B	wild type and *unc-104*	t-test		5.2E-09
S7	B	*unc-6* and *unc-104*	t-test		6.9E-01
S7	B	*unc-40* and *unc-104*	t-test		6.9E-01
